# Phaeophyceaean (Brown Algal) Extracts Activate Plant Defense Systems in *Arabidopsis thaliana* Challenged With *Phytophthora cinnamomi*

**DOI:** 10.3389/fpls.2020.00852

**Published:** 2020-07-07

**Authors:** Md Tohidul Islam, Han Ming Gan, Mark Ziemann, Hashmath Inayath Hussain, Tony Arioli, David Cahill

**Affiliations:** ^1^School of Life and Environmental Sciences, Deakin University, Waurn Ponds Campus, Geelong, VIC, Australia; ^2^Department of Plant Pathology, Faculty of Agriculture, Sher-e-Bangla Agricultural University, Dhaka, Bangladesh; ^3^Seasol International R&D Department, Bayswater, VIC, Australia

**Keywords:** seaweed, *Ascophyllum nodosum*, *Durvillaea potatorum*, RNA-Seq, *Phytophthora cinnamomi*, *Arabidopsis thaliana*

## Abstract

Seaweed extracts are important sources of plant biostimulants that boost agricultural productivity to meet current world demand. The ability of seaweed extracts based on either of the Phaeophyceaean species *Ascophyllum nodosum* or *Durvillaea potatorum* to enhance plant growth or suppress plant disease have recently been shown. However, very limited information is available on the mechanisms of suppression of plant disease by such extracts. In addition, there is no information on the ability of a combination of extracts from *A. nodosum* and *D. potatorum* to suppress a plant pathogen or to induce plant defense. The present study has explored the transcriptome, using RNA-seq, of *Arabidopsis thaliana* following treatment with extracts from the two species, or a mixture of both, prior to inoculation with the root pathogen *Phytophthora cinnamomi*. Following inoculation, five time points (0−24 h post-inoculation) that represented early stages in the interaction of the pathogen with its host were assessed for each treatment and compared with their respective water controls. Wide scale transcriptome reprogramming occurred predominantly related to phytohormone biosynthesis and signaling, changes in metabolic processes and cell wall biosynthesis, there was a broad induction of proteolysis pathways, a respiratory burst and numerous defense-related responses were induced. The induction by each seaweed extract of defense-related genes coincident with the time of inoculation showed that the plants were primed for defense prior to infection. Each seaweed extract acted differently in inducing plant defense-related genes. However, major systemic acquired resistance (SAR)-related genes as well as salicylic acid-regulated marker genes (*PR1*, *PR5*, and *NPR1*) and auxin associated genes were found to be commonly up-regulated compared with the controls following treatment with each seaweed extract. Moreover, each seaweed extract suppressed *P. cinnamomi* growth within the roots of inoculated *A. thaliana* by the early induction of defense pathways and likely through ROS-based signaling pathways that were linked to production of ROS. Collectively, the RNA-seq transcriptome analysis revealed the induction by seaweed extracts of suites of genes that are associated with direct or indirect plant defense in addition to responses that require cellular energy to maintain plant growth during biotic stress.

## Introduction

Plants have evolved marvelous interactive and adaptive systems to grow in challenging and changing environments, including the activation of plant defense response systems. Plants are continually exposed to adverse conditions in their environment whether they be under cultivation or as part of a natural system. Adverse growing conditions can lead to compromised plant growth, reproduction and productivity, and can be abiotic and biotic factors that may occur simultaneously. Abiotic factors are those such as drought and soil salinity while biotic factors include insect herbivory and disease caused by various pathogens. Employing new ways to activate plant-defense-response systems to counteract adverse factors could be transformative for agriculture and for enhancing biodiverse landscapes.

In this regard plant biostimulants, such as those made from seaweed extracts, are unique. Plant biostimulants are defined by a biological mode of action that utilizes plant mechanisms to provide their benefits such as enhanced tolerance to stresses, enhanced nutrient use and productivity (Brown and Saa, 2015). In Europe, the general principles used to justify plant biostimulant claims highlight that their effect is independent of nutrient content (Ricci et al., 2019). Plant biostimulants are used at low rates of application which differentiates their mode of action from synthetic nutritional fertilizers. The low dosage range is consistent with plant biostimulants having properties that accentuate plant response systems for better plant growth and improved tolerances. Many published studies have shown that biostimulants provide a multitude of plant growth benefits such as improved tolerances to abiotic and biotic stresses (Khan et al., 2009; Calvo et al., 2014). To achieve such a wide range of plant benefits across diverse plant families, implies that the molecular mechanisms underlying the plant responses are conserved, complex and pleotropic in character. Despite these insights their modes of action remain elusive.

Seaweed extracts are used successfully to improve agricultural productivity (Calvo et al., 2014; Arioli et al., 2015). A greater understanding of their biological modes of action will further enhance productivity gains in the future. There are a range of commercial seaweed-based products which are available off-the-shelf for commercial and home garden care and the majority of these claim that their use promotes plant growth, improves soil quality and/or enhances resistance against biotic and abiotic stress (Arioli et al., 2015). The effect of several of these products on abiotic and biotic stress mitigation and their mechanism of action has been explored in various studies (Khan et al., 2009; Shukla et al., 2019).

Our hypothesis was that plants treated with seaweed extract would increase their tolerances to subsequent stresses through the activation of a combination of plant defense responses. We envisaged this type of mode of action could be extended by combining different types of seaweed extracts.

The conditioning of plants to stress is an important feature for enhancing crop resilience and reducing productivity losses due to abiotic and biotic stresses (Kerchev et al., 2019). Plant conditioning is based on the molecular activation and priming of plant molecular defense systems so enhanced plant tolerance is exhibited upon subsequent stress occurrences (Martinez-Medina et al., 2016). Importantly, pre-treatment of plants using seaweed extracts is a practical approach to proactively initiate the conditioning phenomenon and was incorporated in our experimental design.

This study used a unique combination of approaches for new insights into the effect of different types of seaweed extracts on the activation of plant defense systems. Here, we used three different seaweed extracts, different plant response time points for assessments, a plant pre-treatment approach (to apply the different seaweed extracts) and the well-studied model system of *Arabidopsis thaliana* with the root pathogen *Phytophthora cinnamomi.* Three seaweed-derived extracts, namely extracts from the brown algae *Durvillaea potatorum* (native to the southern hemisphere) and *Ascophyllum nodosum* (native to the northern hemisphere), were used either separately or in a mixture, to treat Arabidopsis plants and then to compare activation of their plant defense systems. The model system of *Arabidopsis thaliana* with the root pathogen *Phytophthora cinnamomi* was utilized to trigger and synchronize the abiotic attack.

*Phytophthora cinnamomi* is an oomycete pathogen with an extremely wide host range. It is a notoriously aggressive forest pathogen and is considered a major threat to natural ecosystems in Australia (Cahill et al., 2008; Hardham and Blackman, 2018; Costa et al., 2020). *P. cinnamomi* is also a serious threat to horticultural, ornamental and nursery industries and, for example, causes one of the most damaging diseases of avocado. The pathogen infects the feeder roots and often the trunk of larger species causing disease that leads to branch die-back, loss of production and eventual death (Reeksting et al., 2016).

To characterize the plant defense response systems upon pathogen inoculation we used molecular and cellular techniques. High throughput RNA-sequencing was used at key plant response time points. This approach complimented the excellent transcriptomics reports on the action of biostimulant extracts (Nair et al., 2012; GonñI et al., 2016; Jithesh et al., 2019; Omidbakhshfard et al., 2020), particularly with respect to the ability of biostimulants to alter the outcome of root pathogen infection. To further confirm the defense transcriptome induction, Reactive Oxygen Species (ROS) were investigated by staining for hydrogen peroxide in the seaweed extract treated plants at 12 h post-inoculation (hpi) with *P. cinnamomi*. Microscopic analysis was performed to confirm that the pathogen had infected the inner root cell layers, and the extent of infection quantified using quantitative PCR. A plate assay was used to confirm that the seaweed extracts had no direct effect on pathogen growth.

We report that the extracts from two different brown seaweeds and their combination, activated plant defense responses upon pathogen-induced stress. Plants treated with each extract had different but overlapping transcriptomic gene expression profiles, and showed higher ROS levels that coincided with the activation of plant defense.

## Materials and Methods

### *Arabidopsis thaliana* Growth Conditions

Seeds of *Arabidopsis thaliana* ecotype Ler (LEHLE, TX, United States^1^) were surface-sterilized within a 1.5 mL microcentrifuge tube that contained 50% v/v ethanol (Chem-supply, Australia), 5% of H_2_O_2_ 30% solution (Sigma-Aldrich, Australia) for 5 min and subsequently rinsed three times in sterile distilled water (sdH_2_O) and suspended in 0.2% (w/v) water agar. The seed suspension was stored in the dark at 4°C for 2−3 days. The stratified seeds were then seeded into Petri dishes (9-cm-diameter) containing Murashige and Skoog basal medium 0.44% (w/v) (Sigma-Aldrich, Australia) supplemented with 3% sucrose (w/v) (Chem-supply, Australia) and 0.8% (w/v) bacteriological agar and adjusted to pH 5.7 with 1 M potassium phosphate dibasic or potassium phosphate monobasic (Sigma-Aldrich, Australia). Seeds were evenly distributed in Petri dishes by placing individual seeds on the agar surface with a 1000 μL pipette tip, 120 seeds per plate. Petri dishes were transferred to a plant growth chamber (Thermoline Scientific, Australia) under cool white fluorescent lights (100 μmol photons m^–1^ s ^–1^) with a 16:8 h (day: night cycle) at 21 ± 2°C for 14 days. Plants of uniform size were then selected for further use in experiments.

### Plant Growth and Treatment With Seaweed Extracts

Plants were grown in a sand-based tube system that used commercial propagation sand (Bunnings, Australia) that was autoclaved and sterilized before adding to the tubes. The tubes used were 5 mL plastic disposable pipette tubes (Axygen, Australia) with a piece of cotton wool (Woolworths, Australia) inserted into the narrow end to form a plug that held the sand in place. Each tube was filled with sand to within 5 mm of the top and then 1 mL of diluted (1:400) seaweed extract or water as the control, was added at the top of the tube to just moisten the sand. The seaweeds used in this study are different, so the extracts are not identical, therefore we standardized the testing approach. The 1:400 dilution of the extracts were chosen because of (i) greenhouse and field studies demonstrating the efficacy for this dilution (Mattner et al., 2013, 2018), and (ii) by testing for root growth efficacy using the dilution as described previously (Arioli et al., 2015). For alignment with our previous greenhouse and field studies and the root growth testing, each of the seaweed extracts where standardized to 16% (w/w) soluble solids before preparing the 1 in 400 dilution for each seaweed extract. Three seaweed extracts designated as “AN” (an alkaline hydrolysis product from *Ascophyllum nodosum*), “DP” (an alkaline hydrolysis product from *Durvillaea potatorum*) and “AN/DP” (an alkaline hydrolysis product from both *A. nodosum* and *D. potatorum, Seasol^TM^*) were used in this study. Single plants of *A. thaliana* seedlings were gently removed from the MS plate and the roots carefully placed within a 10 mm deep hole made by pushing the narrow end of another 5 mL tube into the sand. Following placement of the seedling roots within the hole a further 1 mL of diluted seaweed extract (1:400) or water was added to enclose the root system by the sand. Tubes were then placed in a plastic rack and transferred to the plant growth chamber under the conditions described in section “*Arabidopsis thaliana* Growth Conditions.” Each day, and up until 6 days after placing the seedlings in the growth chamber, 700 μL of seaweed extract (1:400) was added to each tube or distilled water for the control.

### Infection With *Phytophthora cinnamomi* Zoospores

Zoospores of *P. cinnamomi* were produced according to Islam et al. (2017) and the zoospore density adjusted to 1 × 10^5^ zoospores/mL. Inoculation of the roots of plants grown in tubes took place on day seven whereby 700 μL of the zoospore suspension was carefully dispensed by pipette against the side wall of the plant growth tube just above the sand surface. The inoculated plants (8 plants/replicate/treatment) were then harvested at 0 h (i.e., immediately) and then at 3, 6, 12 and 24 h post-inoculation (hpi). To remove individual plants from a growth tube whilst avoiding damage to the root system a tube was briefly submerged in distilled water held within a container and the tube gently tapped to remove sand and the whole plant. The intact plant was then immediately placed with its roots submerged in water within a square plastic culture dish (10 × 10 cm) and the roots agitated gently to remove residual sand particles. Whole plants were gently and briefly dried on absorbent paper and frozen in liquid nitrogen followed by storage at −80°C. To confirm that roots had been inoculated, roots of eight plants from each treatment were sampled at 24 hpi and placed on PARPH medium (Islam et al., 2017) within 9cm-in-diameter Petri plates and examined for typical *P. cinnamomi* hyphal growth after 72 h incubation at 24°C in the dark. Images of whole root systems were also captured using a digital camera at 7 days after transferring the plants into the sand system and root length measured on individual plants with the aid of imageJ software. Final root growth data represent the mean of three biological replicates (each replicate contained 8 plants) from two independent repeats.

### Gene Expression Analysis by Semi-Quantitative PCR

The plants were grown and treated with seaweed extracts as described in section “*Arabidopsis thaliana* Growth Conditions” and “Plant Growth and Treatment With Seaweed Extracts,” respectively. Then the plants were inoculated and harvested (8 plants for each time point for each treatment) at 0 h and then every 3 h until 9 hpi, as described in section “Infection With *Phytophthora cinnamomi* Zoospores.” Total RNA was isolated from plant tissues using a TRIzol^®^-based RNA extraction system (Life Technologies, United States) according to the manufacturer’s instructions. The quantity of RNA was measured using a Nanodrop^®^ spectrophotometer (Thermo Fisher Scientific, United States) and the ratio (>1.8) of sample absorbance at 260/280 was used to determine the purity of samples. All samples extracted were of high yield and purity. The isolated RNA samples were then treated with DNAse-1 (Invitrogen, Carlsbad, CA, United States) according to the manufacturer’s instructions to remove any residual gDNA. Then the Tetro cDNA synthesis kit (Bioline, Australia) was used to synthesize cDNA from isolated RNA according to the manufacturer’s instructions.

#### Semi-Quantitative RT-PCR Conditions

The expression of genes involved in SA and JA mediated pathogen resistance pathways (*PR1*, *PR5*, *NPR1*, *PDF1.2*, and *THI2.1*) described by Lemarié et al. (2015) were examined using semi-quantitative PCR. The *actin* and *EF-1 alpha* genes were used as internal controls. The primer sequences of *NPR1*, *PDF1.2*, *THI2.1*, and *Actin* were as described by Eshraghi et al. (2011) and the primers for *PR1*, *PR5*, and *EF-1 alpha* were designed using primer3plus (Supplementary Table 1). PCR reactions were performed with GoTaq green master mix (Promega, United States) and each reaction contained 2 μL of cDNA and 0.5 μM of the respective primers. PCR cycles consisted of an initial denaturation step of 3 min at 95°C, followed by repetitions (28−36 cycles, depending on the primer set) of the following three steps: a 30 s denaturation step at 95°C, 30 s annealing step ranging between 54°C and 60°C, 1 min elongation step at 72°C and a final extension step at 72° for 5 min. Initial reactions were performed to determine the annealing temperature of each primer set and the appropriate cycle number of the PCR reaction. PCR products were analyzed on a 1% agarose gel with 0.5 × TBE buffer and visualized using gel red staining with a gel doc system. The final gel images are representative of two biological replicates from two experimental repeats.

### Examination of the Host Transcriptome Using RNA-Seq

#### Plant Growth, Seaweed Extract Treatments, and Plant Inoculation

*Arabidopsis thaliana* plants were grown as described in section “*Arabidopsis thaliana* Growth Conditions” and seaweed extracts were applied as described in section “Plant Growth and Treatment With Seaweed Extracts.” The plants were inoculated as described in section “Infection With *Phytophthora cinnamomi* Zoospores” and harvested at 0, 3, 6, 12, and 24 hpi (8 plants at each time point for each treatment). Control plants, treated with water alone, were also harvested at these times as described in section “Plant Growth and Treatment With Seaweed Extracts.” The harvested plants were immediately frozen in liquid nitrogen and kept in a −80°C freezer until RNA was extracted. This experiment contained three biological replicates at each time point for each treatment (5 time points of four treatments including the control resulted in 60 samples for analysis).

#### RNA Extraction and cDNA Library Preparation

The total RNA was extracted from whole plants using a commercial kit (RNeasy Plant Mini Kit, Qiagen, Valencia, CA, United States) following the manufacturer’s instructions. RNA concentration and integrity was then determined first using spectrophotometry (NanoDrop ND-1000 spectrophotometer, Thermo Fisher Scientific, United States) with absorbance ratio of A260/280 nm and A260/230 nm. RNA integrity was also confirmed with the 4200 TapeStation system (Agilent Technologies, Santa Clara, CA, United States). Only RNA samples with an A260/280 nm ratio between 2.0 and 2.1 and RNA integrity number (RIN) greater than 7 (Sigurgeirsson et al., 2014) were used for further analysis. DNA libraries were constructed from total RNA of control and inoculated samples using the NEBNext^®^ Ultra^TM^ II Directional RNA Library Prep Kit for Illumina^®^ according to the manufacturer’s instructions (New England BioLabs, United States). The quality of the libraries was assessed by using a 4200 TapeStation 6000 system and their quantities were measured by Qubit dsDNA BR assay kit (Thermo Fisher Scientific, United States). The library was sequenced using the NovaSeq 6000 platform (Illumina) in a paired-end 150 bp run.

#### Processing of Sequenced Reads

The sequencer-generated raw reads were pre-processed and mapped to the reference genome using CLC Genomics Workbench (version 8.5.1, CLC Bio, Arhus, Denmark). During pre-processing of RNA-Seq data, adapter sequences, reads with >10% of unknown bases, low quality reads (sequences with more than 50% bases with quality value ≤ 5) and ambiguous bases were removed to obtain high quality reads for further analysis. High quality reads were mapped to the reference *A. thaliana* genome using the default parameters of the Workbench software to generate normalized gene expression values in the form of reads per kilobase of transcript per million mapped reads (RPKM). Proportion-based statistical analysis of differentially expressed reads was performed using default parameters to identify differentially expressed genes (DEGs) between water treated and each seaweed treated sample harvested at different hours post-infection by *P. cinnamomi*. DEGs were then filtered based on a FDR-corrected *P*-value of <0.05 and a fold change of ≥1.5 for up-regulated DEGs and ≤−1.5 for downregulated genes. The Illumina RNA-Seq datasets analyzed for this study have been deposited in the SRA database with the accession number of PRJNA609590^2^.

#### Functional Classification of Up-Regulated DEGs

##### Gene ontology and KEGG

The Blast2go 5 PRO (B2G) program was used to perform GO functional classification into biological process, molecular functions and cellular components to analyze the up-regulated gene function distribution at a macro level. Further, the B2G program was also used for Kyoto Encyclopedia of Genes and Genomes (KEGG) annotations for up-regulated DEGs by searching against the KEGG database^3^.

##### Hierarchical clustering and heatmap visualization

For each treatment type, 30 genes with the largest sum of absolute *t*-test Z-scores were selected. With R version 3.6.2 (R Core Team, 2019), the heatmap.2 function was used to display Z-scores across the five time points. For multidimensional scaling analysis, the uniquely mapped read counts for each gene in each sample underwent library size normalization and distance estimation using the cmd scale function. Hierarchical clustering was performed using the hclust function with Spearman correlation values and a tree cut parameter of 0.67.

##### Pathway analysis

The Gaussian based *t*-test Z statistic (CLC Bio) was used to rank genes from most up-regulated to most down-regulated prior to Gene Set Enrichment Analysis (GSEA) with gene sets obtained from the Reactome and MapMan databases (Thimm et al., 2004; Naithani et al., 2016). Enrichment analysis was performed using the FGSEA R package version 1.12.0 (Korotkevich et al., 2019) with a significance threshold of FDR adjusted *p*-Values less than 0.05.

### Quantitative PCR to Validate RNA-Seq Expression

The Tetro cDNA synthesis kit (Bioline) was used to synthesize cDNA from previously isolated RNA that was used for RNA-seq analysis. Briefly, 1 μg RNA was mixed with 1 μL random hexamer, 1 μL 10 mM dNTP mix (final concentration 0.5 mM), 1 μL ribosafe RNase inhibitor (final concentration 0.5 u/μL), 4 μL 5 × RT buffer, 1 μL tetro reverse transcriptase (final concentration 10 u/μL), and DEPC-treated water up to a total of 20 μL. Then the mix was incubated in the PCR machine according to the following order: initial incubation 25°C for 10 min followed by 45°C for 30 min and then the reaction terminated at 85°C for 5 min.

The primers of all tested genes were designed using primer3plus software (Supplementary Table 2) and annealing temperature of each primer pair was selected using gradient qPCR. The resulting qPCR product was analyzed via gel-electrophoresis to check that the correct gene product was obtained based on the primer design. Moreover, PCR efficiency of all genes was determined by a standard curve analysis of cDNA samples using a series of 10-fold dilutions of cDNA to determine the gene-specific PCR amplification efficiency for each primer pair used in RT-qPCR experiments. The real time PCR amplifications were carried out using SYBR Green detection chemistry. cDNAs were run in triplicate on 96 well reaction plates with the CFX Connect real time PCR (Bio-Rad, United States). 10 μL of reaction mixture containing 5 μL of iTaq^TM^ universal SYBR Green Mix (Bio-Rad, United States), 0.4 μL of each 10 μM of primer and 2 μL of diluted cDNA as template and 2.2 μL RNase/DNase free sterile water (Sigma-Aldrich, Australia). The following amplification program was used in all RT-qPCR reactions: 95°C for 3 min, 40 cycles of 95°C for 10 s and annealing temperature (54−60°C) for 30 s at optimized temperatures for specific candidate genes. The specificity of each amplification reaction was verified by a melting curve analysis after 40 cycles. No template controls (NTC) were included for each primer pair to avoid possible contamination of assay reagents. Three biological replicates were used for each time point and each reaction was run in triplicate for each target and reference gene. All samples were run in parallel with actin reference genes (*ACT2* and *ACT8*) to normalize cDNA loading. The relative expression values for each target gene were calculated against reference genes using the following equation according to Livak and Schmittgen (2001): ΔΔ*C*T = (*C*T, Target – *C*T, reference gene) Time X − (*C*T, Target – *C*T, reference gene) Time 0.

### Microscopic Examination of *P. cinnamomi* Infection of Roots of *A. thaliana*

To monitor the root infection process following inoculation with motile zoospores, the plants were grown with seaweed extracts or water as a control and inoculated with *P. cinnamomi* as described in section “*Arabidopsis thaliana* Growth Conditions,” “Plant Growth and Treatment With Seaweed Extracts,” and “Infection With *Phytophthora cinnamomi* Zoospores.” Then the plants were harvested at 12, 24, 48, and 72 hpi for each extract treatment and the roots removed and stained using a trypan blue staining protocol (Wang et al., 2011). Briefly, harvested roots were transferred into 2 mL Eppendorf tubes containing diluted trypan blue solution (10 g phenol, 10 mL glycerol, 10 mL lactic acid, 10 mL water and 10 mg of trypan blue). The tubes were incubated in a heated water bath and boiled for 4 min. After cooling to room temperature, the samples were de-stained by replacing the staining solution with chloral hydrate solution (5 g chloral hydrate/2 mL water) for 24 h. The samples were finally mounted in 50% glycerol and viewed with a light microscope (Zeiss, Göttingen, Germany) using bright field illumination. Images were captured with a digital camera (Zeiss, Göttingen, Germany) mounted on the microscope. The final images are representative of three biological replicates (each with at least 5 plants) at each time point for each treatment.

### *P. cinnamomi* Quantification Using qPCR

To quantify the amount of *P. cinnamomi* in *A. thaliana* roots following inoculation plants were first grown with seaweed extracts (AN, DP, and AN/DP) for 6 days and inoculated with *P. cinnamomi* as described in section “*Arabidopsis thaliana* Growth Conditions,” “Plant Growth and Treatment With Seaweed Extracts,” and “Infection With *Phytophthora cinnamomi* Zoospores.” Then, the plant roots were harvested at 12, 24, 48, 72, and 96 h post-inoculation (hpi). The pathogen was quantified from harvested roots according to Engelbrecht et al. (2013). Briefly, DNA from a *P. cinnamomi* culture was extracted using PrepMan Ultra Reagent (Thermo Fisher Scientific) according to the manufacturer’s protocol and kept at −20°C until further use. DNA from root samples was extracted using the CTAB based method (Supplementary Method 1). The amount of plant genomic DNA present within the sample was quantified first by real-time PCR using primers (Supplementary Method 1) amplifying the A. *thaliana* a*ctin* gene. A normal one-step real-time PCR was conducted for the plant a*ctin* gene. The amount of plant DNA was calculated using a standard curve developed from a series of known concentrations of *A. thaliana* genomic DNA. The amount of *P. cinnamomi* DNA present within *A. thaliana* root samples was quantified using a nested real-time PCR method. LPV3 primers (Engelbrecht et al., 2013) were used in the outer first round of PCR, then LPV3N primers were used for the second round nested PCR to bind within the outer PCR product (Supplementary Method 1). The amount of pathogen DNA was calculated based on a standard curve developed from a series of known concentrations of *P. cinnamomi* DNA. Finally the quantity of *P. cinnamomi* was determined as ng of *P. cinnamomi* DNA/100 ng of *A. thaliana* DNA. The final amount determined represents the mean of three biological replicates from two independent experimental repeats.

### Histochemical Localization of Hydrogen Peroxide (H_2_O_2_)

For hydrogen peroxide (H_2_O_2_) detection, the plants were grown and inoculated as described in section “*Arabidopsis thaliana* Growth Conditions,” “Plant Growth and Treatment With Seaweed Extracts,” and “Infection With *Phytophthora cinnamomi* Zoospores”. At 12 hpi, the whole plants were harvested and were placed in a 2 mL micro centrifuge tube with 1 mL diaminobenzidine (DAB) (1 mg/mL). The seedlings were incubated in the dark at room temperature for 3 h for H_2_O_2_ detection (Thordal-Christensen et al., 1997;Venus and Oelmüller, 2013). Samples were then transferred to and incubated in a decoloring solution (EtOH: lactic acid: glycerol = 1:1:1) at 80°C for 20 min (Venus and Oelmüller, 2013). Seedling roots were then visualized using a light microscope (Zeiss, Göttingen, Germany) with bright field illumination and images captured with a digital camera mounted on the microscope. The optical density of the colored precipitate was measured using imageJ software. The final images were representative of three biological replicates each with 5 plants.

## Results

### The Influence of Seaweed Extracts on *A. thaliana* Root Growth and Infection by *P. cinnamomi*

*Arabidopsis thaliana* Ler seedlings were grown in a sand growth system (distilled water as a control and 1:400 seaweed extract as a treatment) and root lengths were measured 7 days after the commencement of treatment. All three seaweed extracts (AN, DP, and AN/DP) significantly enhanced root growth compared with the controls (Supplementary Figure 1). Moreover, root growth was observed to be significantly higher in extract-treated plants at the time of inoculation and the difference in root growth rate was maintained up to 96 hpi (Data not shown).

**FIGURE 1:**
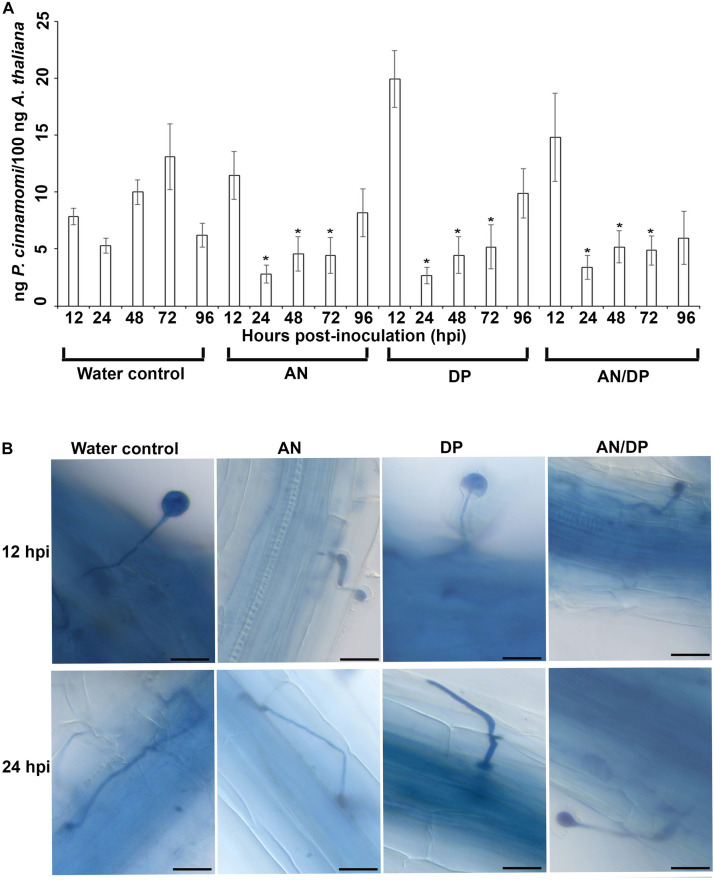
Effect of seaweed extracts on *P. cinnamomi* infection in roots of *A. thaliana*. Plants were grown in a sand culture system with either seaweed extracts (AN, DP, and AN/DP) or water as a control for 6 days and then inoculated with *P. cinnamomi* on day 7. **(A)** Nested real time PCR quantification of *P. cinnamomi* DNA. Plant roots were harvested from 12 to 96 hpi. Data presented are from two experimental repeats each with three biological replicates. Error bars represent the standard error of means. *significant difference for amount of pathogen in different treatment compared to water control at *P* = 0.05 according to Duncan’s multiple range test. **(B)** Whole roots of *A. thaliana* infected with *P. cinnamomi* zoospores following treatment of roots with seaweed extracts (AN, DP, or AN/DP). Images were captured at 12 and 24 hpi. Scale bar = 20 μM. Each image is representative of three biological replicates.

The quantitative measurement of the amount of *P. cinnamomi* in *A. thaliana* roots grown with seaweed extracts showed that overall there was less pathogen growth compared with the water control at 24, 48, and 72 hpi (Figure 1A). However, at 12 hpi, the amount of *P. cinnamomi* was higher in all three seaweed extract-treated roots compared to the water control. For the AN and DP seaweed extract- treated roots about the same amount of pathogen was found at 96 hpi, as was found in the water treated controls. Notably the combined extract (AN/DP) showed a plateauing of the amount of *P. cinnamomi* from 24 h onward to a level that was sustained well below that of the controls (Figure 1A).

### Analysis of SA and JA Related Gene Expression

The expression of SA and JA-related genes, *PR1*, *PR5*, *NPR1*, *PDF1.2* and *THI2.1* was analyzed using semi-quantitative PCR and the *Actin* gene was used as an internal control to confirm even loading of DNA and reaction efficiencies of all cDNA samples prepared. A differential expression pattern was found for each resistance-related gene in all three seaweed extract treatments. Results showed that *Actin* expressed equally in all tested cDNA samples indicating the quality of cDNA and equal loading on the gel (Supplementary Figure 2). A higher expression of *PR1* was found in AN-treated plants at 3 hpi and the expression increased at 6 and 9 hpi. A similar trend was found for the AN/DP treatment. However, consistently higher expression from 3 hpi was observed for the DP treatment. A similar expression pattern (up-regulated at 3 hpi) was recorded for *PR5*. Moreover, the expression of *NPR1* was found to be consistently induced in all three treatments. The expression of *PDF1.2* was found to be higher only in those plants treated with DP and AN/DP. In addition, the expression of *THI2.1* was up-regulated at 3 hpi in plants treated with AN, and at 6 and 9 hpi in those plants treated with DP and AN/DP (Supplementary Figure 2).

### Transcriptome Analysis of Plants Treated With Seaweed Extracts and Then Infected With *P. cinnamomi*

#### Confirmation of Pathogen Infection in Roots

Microscopic examination of *A. thaliana* roots grown with seaweed extracts and inoculated with *P. cinnamomi* revealed the different patterns of penetration and establishment of infection (Figure 1B). This microscopic analysis confirmed that the system established and optimized for this study was one in which the plants were successfully inoculated and that the pathogen grew both on the root surface and within the root.

#### Overview of RNA-Seq Data and Mapping to the *A. thaliana* Reference Genome

The Nova-Seq platform generated 43−155 million reads (average length 151 bp, paired end reads) and these reads were processed through CLC genomics workbench to remove adapters and ambiguous reads from the samples. A read refers to the sequence of a cluster that is obtained after the end of the sequencing process which is ultimately the sequence of a section of a unique fragment. After trimming, more than 98% of reads were recovered as high quality reads to proceed for mapping to the reference genome (Table 1).

**TABLE 1:** RNA-Seq read statistics before mapping and after quality selection and trimming.

Sample id	Total reads	Total nucleotides	Total reads after trimming	Percentage of reads after trimming
**Water control**				
H-0	54,233,072	7,492,370,850	53,448,230	98.22
H-3	51,132,182	8,447,132,374	50,478,514	98.70
H-6	107,420,061	16,220,429,261	105,873,930	98.64
H-12	51,528,171	6,934,219,584	51,256,437	99.27
H-24	52,952,768	7,824,829,664	51,259,041	96.95
**AN treatment**				
AN-0	66,404,596	10,027,093,996	65,450,105	98.62
AN-3	137,378,291	20,744,121,891	135,219,054	98.24
AN-6	152,472,398	23,023,332,098	149,964,428	98.49
AN-12	60,409,115	9,121,776,415	59,615,072	98.68
AN-24	155,067,084	23,415,129,684	153,620,985	99.33
**DP treatment**				
DP-0	47,324,764	7,146,039,364	47,164,165	99.64
DP-3	48,885,610	7,377,197,110	48,824,421	99.93
DP-6	45,391,111	6,854,057,711	45,343,642	99.89
DP-12	44,783,657	6,762,331,553	44,719,573	99.87
DP-24	46,672,822	7,047,596,122	46,627,376	99.90
**AN/DP treatment**				
AN/DP-0	43,800,423	6,613,863,823	43,421,661	99.23
AN/DP-3	49,173,252	7,425,161,052	49,052,075	99.80
AN/DP-6	48,539,686	7,328,092,586	48,140,503	99.20
AN/DP-12	78,530,707	11,858,136,807	78,425,815	99.87
AN/DP-24	65,967,493	9,961,091,393	78,425,815	99.72

*Data are presented as the average of three biological replicates.*

On average, more than 87% of the total reads were mapped to the reference *A. thaliana* genome. The reads not mapped to the reference genome were expected to be pathogen reads as the samples were inoculated with *P. cinnamomi*. In addition, there were few reads that were mapped as broken pairs (Supplementary Table 3).

#### Overview of Differentially Expressed Genes (DEGs)

Differentially expressed genes (DEGs) were identified based on their expression value normalized through Reads Per Kilobase per Million mapped reads (RPKM) according to a previous study (Kasirajan et al., 2018). RPKM estimates the gene expression level of a gene normalized for both transcript length and library sequencing depth, allowing a direct comparison of expression levels within and between samples. The following parameters were considered to filter the DEGs of each treatment: FDR corrected *P-*value ≤ 0.05, fold change ≥ 1.5 for up-regulated genes, fold change ≤ 1.5 for down-regulated genes, each sample was compared with the respective water control. The highest number of up-regulated genes was found in the AN-treated samples harvested at 12 hpi followed by the AN/DP-treated samples harvested at 24 hpi. The highest number of down-regulated genes was found in the AN/DP-treated samples harvested at 3 hpi followed by those harvested at 6 hpi (Supplementary Table 4).

#### Functional Analysis of Up-Regulated DEGs

##### Comparison of number of DEGs between treatments and time points

**FIGURE 2:**
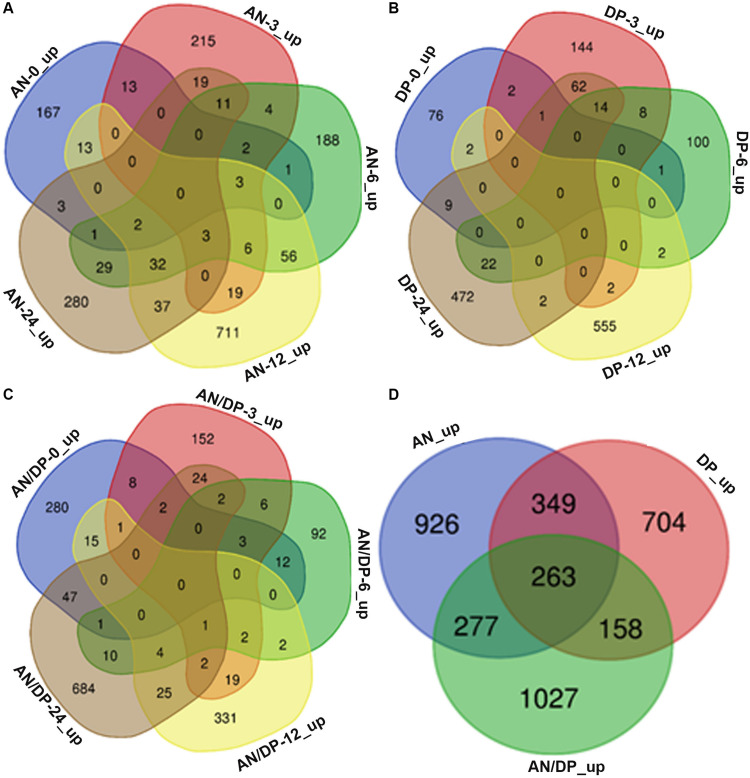
Venn diagrams that show commonalities and differences among up-regulated DEGs at five time points following treatment of *A. thaliana* with **(A)** AN, **(B)** DP, **(C)** AN/DP, and **(D)** up-regulated DEGs (at at least one time point) for each of the three treatments.

Venn diagram analysis revealed that most of the DEGs from each of the three treatments were uniquely expressed at a specific time point (Figures 2A–C). For example, a total of 882 DEGs were found to be expressed in the AN treatment harvested at 12 hpi and among them 711 DEGs were uniquely expressed at this time point. Whereas there were 56 DEGs that were common to the 6 hpi time point, 37 DEGs were commonly expressed at the 24 hpi time point, 19 DEGs were commonly expressed at the 3 hpi time point and 13 DEGs at the 0 hpi time point.

Figure 2D shows that a total of 3,704 unique DEGs (expressed at least at one time point, filtered according to the criteria mentioned above) were found in the three treatments. Out of them, 926, 704 and 1027 were expressed in the AN, DP and AN/DP treatments, respectively. Moreover, 349 DEGs were commonly expressed in the AN & DP treatments whereas 277 DEGs were common to the AN & AN/DP treatments and 158 DEGs were common to the DP & AN/DP treatments. In addition, 263 DEGs were commonly up-regulated across all three treatments.

##### Gene ontology (GO)

The functional classification of up-regulated DEGs was analyzed using Gene Ontology (GO) and classified into three broad categories: molecular function, biological process and cellular component. These broad categories are very useful for identifying the key changes brought about by the treatments.

**FIGURE 3:**
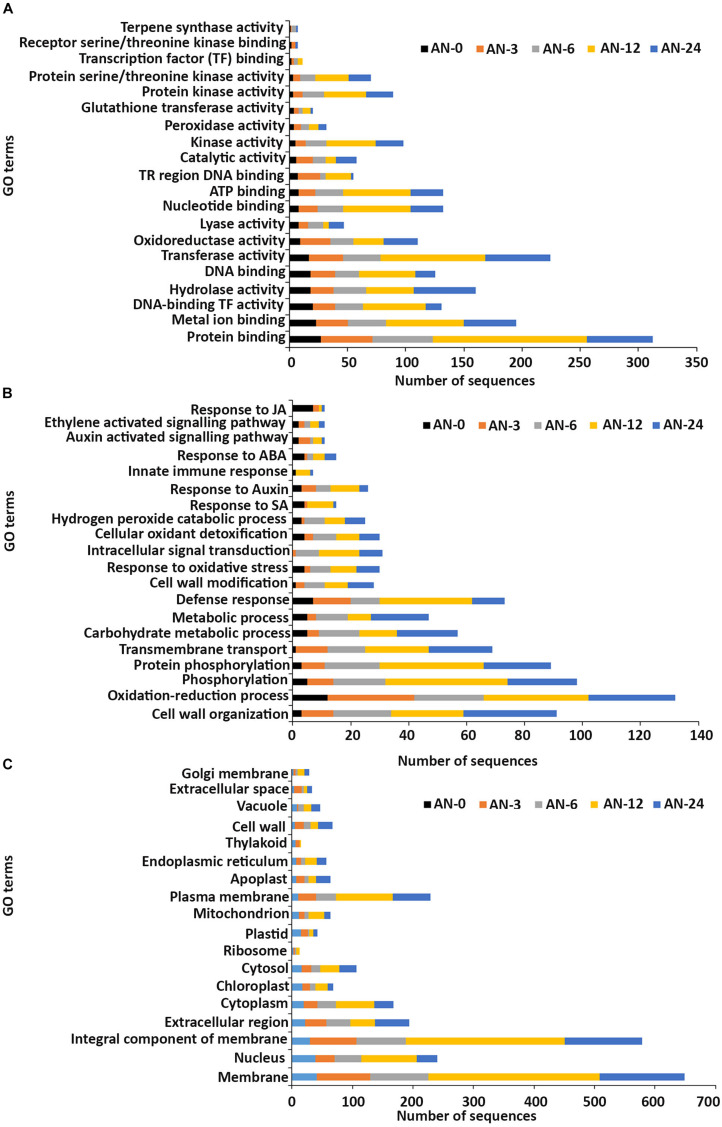
Gene ontology (GO) enrichment analysis of differentially expressed genes (DEGs) from the AN treatment. The DEGs were categorized into panels **(A)** Molecular function, **(B)** Biological process, and **(C)** Cellular component.

**FIGURE 4:**
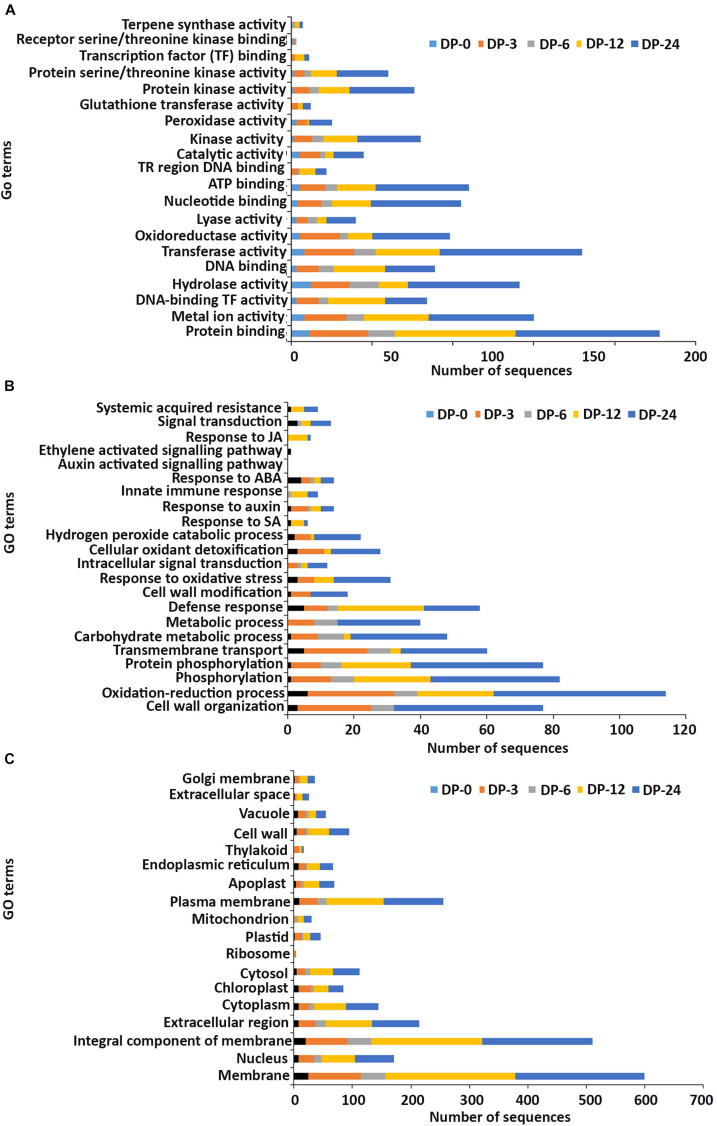
Gene ontology (GO) enrichment analysis of differentially expressed genes (DEGs) from the DP treatment. The DEGs were categorized into panels **(A)** Molecular function, **(B)** Biological process, and **(C)** Cellular component.

**FIGURE 5:**
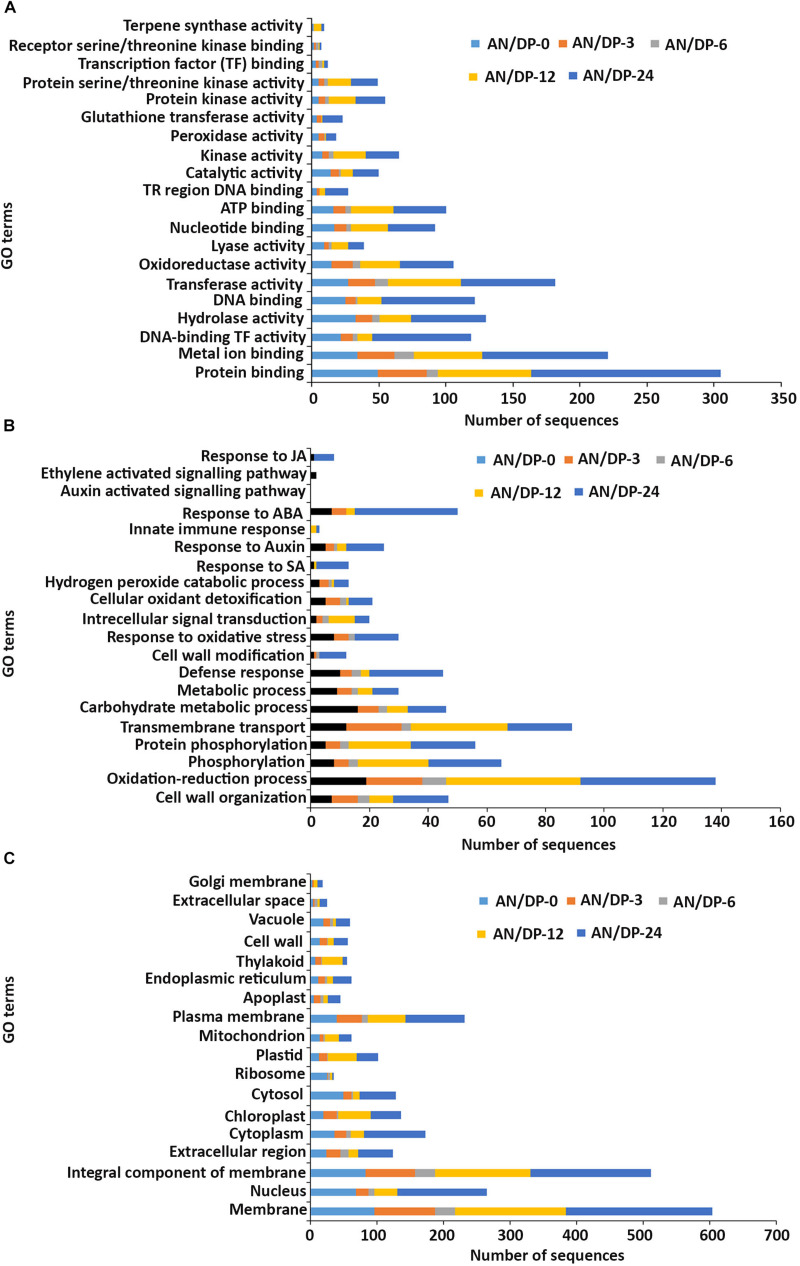
Gene ontology (GO) enrichment analysis of differentially expressed genes (DEGs) from the AN/DP treatment. The DEGs were categorized into panel **(A)** Molecular function, **(B)** Biological process, and **(C)** Cellular component.

In the **molecular function** GO category, protein-binding and metal ion-binding were highly represented for all three treatments (Figures 3–5). Most importantly, the categories associated with plant defense pathways such as hydrolase activity, kinase activity, protein serine/threonine kinase activity, transcription factor binding, receptor serine/threonine kinase binding and terpene synthase activity were enriched in the analysis. For example, protein kinases play a central role in signaling in pathogen recognition and the subsequent activation of plant defense mechanisms (Romeis, 2001). Moreover, the genes identified as having hydrolase activity are likely to be involved in hydrolyzing the pathogen cell wall (Serrazina et al., 2015). The highest number of genes that were represented in the different molecular function categories was found at the 12 hpi time point for the AN treatment whereas for the other two treatments it was at the 24 hpi time point (Figures 3–5).

Most of the up-regulated transcripts in all three seaweed extract-treated *A. thaliana* plants fell into the **biological process** categories of cell wall organization, oxidation-reduction process and phosphorylation (Figures 3–5). In terms of the most important categories related to plant defense pathway processes the following were identified: defense response, hydrogen peroxide catabolic process, response to salicylic acid, response to auxin, innate immune response, response to abscisic acid, auxin activated signaling pathway, ethylene-activated signaling pathway and response to jasmonic acid, all were enriched in the biological process category. Classical defense phytohormones such as salicylic acid (SA), jasmonic acid (JA), ethylene (ET) and more recently, growth-related phytohormones, such as auxins, cytokinins (CKs), brassinosteroids (BRs), abscisic acid (ABA), and gibberellins (GAs) have all been shown to modulate plant immune defenses (Han and Kahmann, 2019).

**FIGURE 6:**
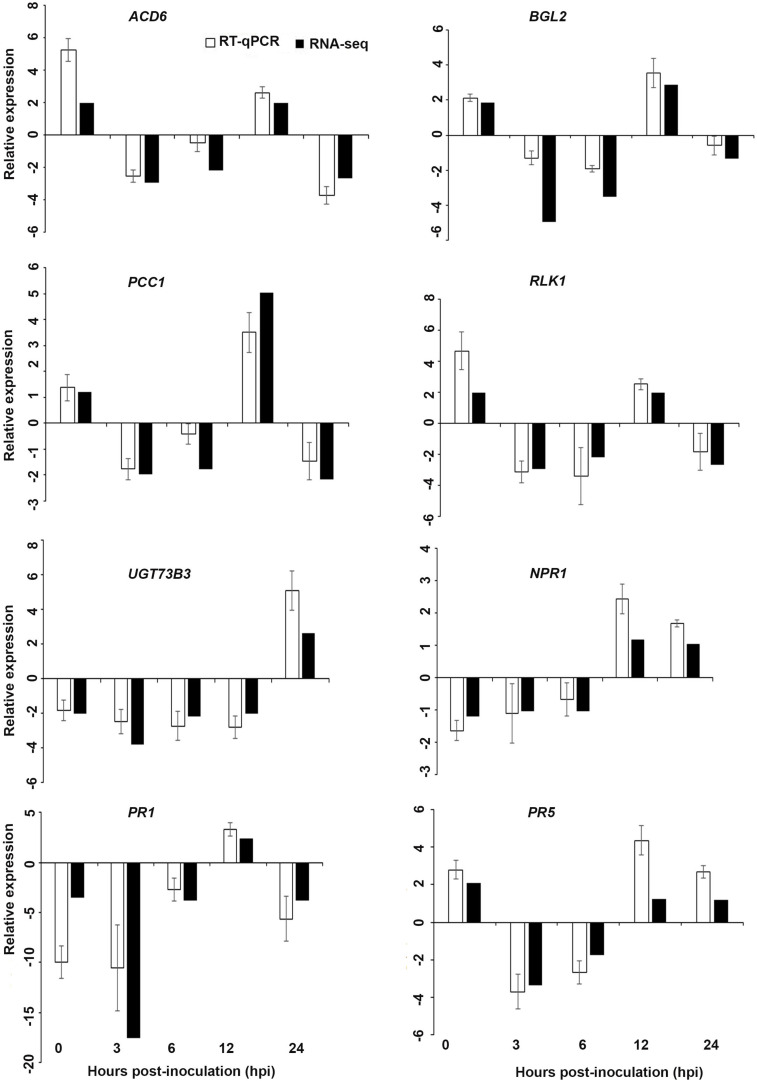
Validation of the differentially expressed genes by RT-qPCR for *A. thaliana* plants treated with AN extract. Samples were collected from the plants grown with the seaweed extract and harvested at 0, 3, 6, 12, and 24 h after *P. cinnamomi* inoculation. All data were normalized to the expression level of *actin 2* (*ACT2*) and *actin 8* (*ACT8*). The data represent the fold change at each time point in the infected samples vs. the control sample. Bars show the standard error of the mean from three biological replicates.

**FIGURE 7:**
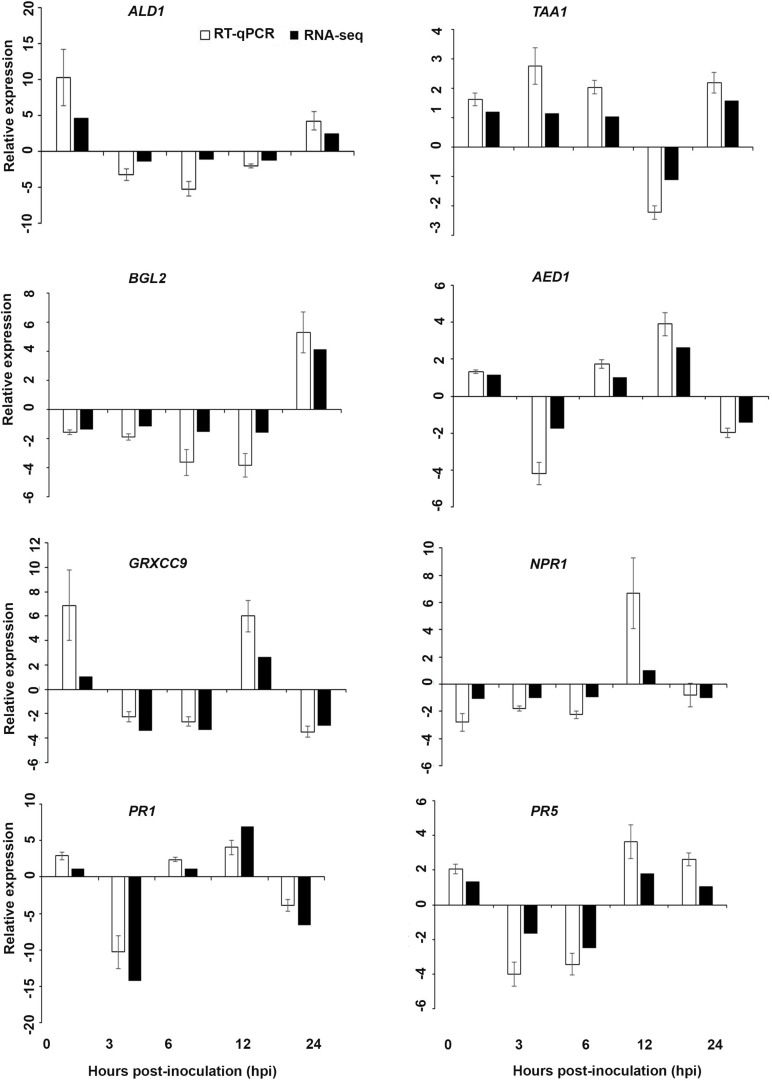
Validation of the differentially expressed genes by RT-qPCR for *A. thaliana* plants treated with the DP extract. Samples were collected from the plants grown with the seaweed extract and harvested at 0, 3, 6, 12, and 24 h after *P. cinnamomi* inoculation. All data were normalized to the expression level of *actin 2* (*ACT2*) and *actin 8* (*ACT8*). The data represent the fold change at each time point in the infected samples vs. the control sample. Bars show the standard error of the mean from three biological replicates.

**FIGURE 8:**
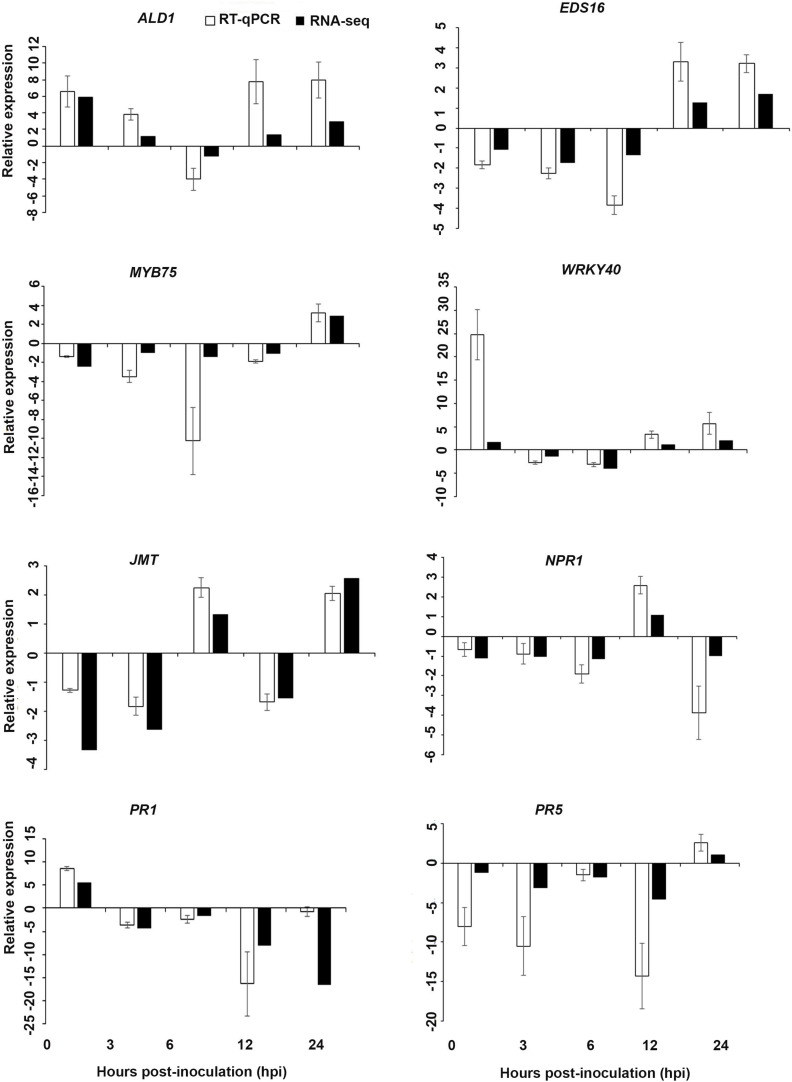
Validations of the differentially expressed genes by RT-qPCR for *A. thaliana* plants treated with the AN/DP extract. Samples were collected from the plants grown with the seaweed extract and harvested at 0, 3, 6, 12, and 24 h after *P. cinnamomi* inoculation. All data were normalized to the expression level of *actin 2* (*ACT2*) and *actin 8* (*ACT8*). The data represent the fold change at each time point in the infected samples vs. the control sample. Bars show the standard error of the mean from three biological replicates.

Interestingly, in a comparison between the three treatments, an up-regulation of the expression of genes related to systemic acquired resistance was found only in the DP treatment at different time points after inoculation (Figures 3–5). However, the induction of expression of three major SAR genes (*PR1*, *PR5* and *NPR1*) was found in the qPCR validation of the RNA-Seq results (Figures 6–8).

In the **cellular component** GO category, up-regulated DEGs in all three of the seaweed extract-treated and inoculated plants were principally assigned to the categories membrane, nucleus, integral component of membrane, plasma membrane and extracellular region (Figures 3–5). A similar predominance of these GO categories was also found in the resistance of plants to incompatible pathogens (Song et al., 2019). For example, cell surface receptors are *trans*-membrane proteins that bind signal molecules in the extracellular space and generate different intracellular signals on the opposite side of the plasma membrane (Alberts et al., 2014).

##### KEGG pathway analysis

Kyoto Encyclopedia of Genes and Genomes analysis showed that different biosynthetic and metabolic pathways were up-regulated in response to *P. cinnamomi* infection of *A. thaliana* grown with the seaweed extracts. The most highly represented top five pathways that contained the largest numbers of up-regulated genes were purine metabolism, biosynthesis of antibiotics, thiamine metabolism, starch and sucrose metabolism and phenylpropanoid biosynthesis. The up-regulation of gene expression related to antibiotic biosynthesis in all three treatments revealed that the plant may use “antibiotic compounds” to combat the pathogen. Moreover, several genes were up-regulated that are associated with phenylalanine metabolism and terpenoid backbone biosynthesis which lead to the synthesis of antimicrobial phytoalexins, phytoanticipins and phenolic compounds that are known to be involved in plant defense against pathogens (Cho and Lee, 2015; Supplementary Figure 3).

##### Clustering and heatmap visualization of DEGs

To visualize the expression pattern of DEGs we performed a hierarchical clustering of the DEGs that were extracted at each time point for each treatment and the respective control. The clustering heatmap (Supplementary Figure 4) showed a complex pattern of gene expression at each time point for each treatment compared to the water control. The heatmap showed that the expression pattern of the following groups were similar: DP-12 and DP-24, AN/DP-3 and AN/DP -6, AN/DP -0 and DP-6, AN/DP -12 and AN-12, H-6 and AN-6 (Supplementary Figure 4). Further, we visualized the top 30 DEGs across the time points for each treatment. The result showed that most of the genes were up-regulated at 12 hpi followed by 24 hpi for the AN treatment. Moreover, *WRKY42* and *CML8* showed the most consistent up-regulation across the time series (Supplementary Figure 5). However, for those genes that were up-regulated most were only up-regulated at 12 and 24 hpi for the DP treatment (Supplementary Figure 6). In addition, the expression patterns of selected genes were slightly different for the AN/DP treatment where the genes were found to be more highly up-regulated at each time point except at 3 hpi (Supplementary Figure 7).

##### Gene set enrichment analysis (GSEA) using Reactome and MapMan databases

The list of DEGs were mapped using GSEA to Reactome and MapMan databases to reveal any pathways that contained a large proportion of genes. The GSEA categorized the DEGs at each time point for each treatment into a number of functional groups. Among those groups obtained from the Reactome database for the AN treatment batch, auxin signaling was dominant at all time points. Also, ABA- and ET-associated genes were enriched at various time points. In addition, a number of important categories related to plant defense reactions such as SA signaling, recognition of fungal and bacterial pathogen and immunity responses were dominant at 12 hpi for the AN treatment (Supplementary Figure 8). Similarly, auxin signaling was dominant at 0 hpi as well as at the early infection stages (6 and 24 hpi) for the DP treatment. The steroid phytohormone, i.e., brassinosteroids-group was enriched at 3, 6, and 24 hpi for DP-treated plants. Most importantly, the SA signaling was dominant at 12 hpi for the DP-treated plants. In addition, the ethylene signaling group was found to be represented at only 6 and 24 hpi (Supplementary Figure 9). The ABA and ET signaling and biosynthesis groups were dominant at 0 hpi and at the early infection time of 3 hpi for the AN/DP treatment whereas brassinosteroids, SA and secondary metabolism groups were found at 3 and up to 12 hpi. The auxin signaling group was found to be dominant at 12 hpi in the AN/DP-treated plants (Supplementary Figure 10).

The different GSEA categories defined using the MapMan databases of DEGs from different treatments are presented in Supplementary Figures 11–13. The biotic stress_PR proteins_Plant defensins group was dominant at 0 and 3 hpi in the AN treatment whereas the biotic stress- associated group was found at later time points (6−24 hpi). The calcium signaling, protein degrading serine proteases, signaling G-proteins and signaling MAP kinases dominated at different time points. Most importantly, secondary metabolism of phenylpropanoids and peroxidases were highly represented at 12 hpi for the AN treatment (Supplementary Figure 11). Similar categories were also found for the DP treatment. However, protein degrading aspartate proteases, BHLH transcription factor and transcription regulator categories were found at different hpi for the DP treatments. A BZIP transcription factor category was highly dominant at 12 hpi for the DP treatment (Supplementary Figure 12). Many of these categories were also similarly found in AN/DP treatments at different hpi, except for 24 hpi where biotic stress was dominated by metabolite transporter, leucine-rich repeat signaling receptor kinases and MYB transcription factor family proteins (Supplementary Figure 13).

**FIGURE 9:**
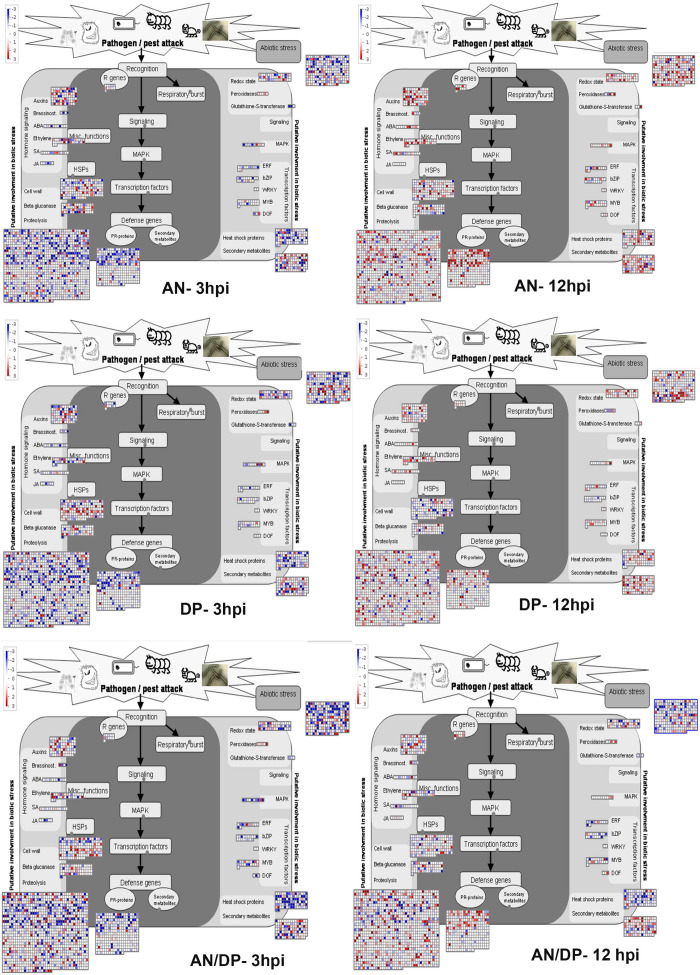
Mapman overview of DEGs related to hormone, stress and metabolic responses in plants of *A. thaliana* following seaweed treatment and after inoculation with *P. cinnamomi* (at 3 and 12 hpi). The average fold change of genes are indicated by the color scale (red represents up-regulated genes and blue represents down-regulated genes).

Understanding the expression pattern of important stress-related genes at different time points following infection with the pathogen is necessary for pinpointing their specific contribution to plant defense. A closer look at the MapMan profile in regards to biotic stress pathways affected by each seaweed extract treatment clearly showed that R genes, proteolysis, cell wall, beta glucanase, phytohormones, respiratory burst, heat shock proteins, secondary metabolites and transcription factor- associated genes were up-regulated at all time points for each treatment (Figure 9 and Supplementary Figure 14). In terms of phytohormones, Auxin, BRs, SA, ABA and ET-associated genes were represented and up-regulated at most of the time points for each treatment. However, JA associated genes were only up-regulated in the AN treatments at 6 hpi. In terms of respiratory burst, redox state- and peroxidases-associated genes were induced in all treatments. However, glutathione-S-transferase (GST) was only up-regulated at 12 hpi for both the AN and DP treatments and at 24 hpi for treatment with AN/DP. Importantly, the greatest number of genes in each category were found to be up-regulated at 12 and 24 hpi for each extract treatment (Figure 9 and Supplementary Figure 14).

#### Validation of RNA-Seq Expression

Based on their known involvement in plant defense pathways five genes from the DEG list for each treatment and three SAR-related genes were selected based on their initial gene expression analysis (see section “Analysis of SA and JA Related Gene Expression”) for RT-qPCR using their specific primers to confirm the reliability of expression of DEGs obtained from RNA sequencing. The relative expression levels of the selected genes were determined at 0, 3, 6, 12, and 24 h after *P. cinnamomi* inoculation. All of the selected genes from the three seaweed extract treatments showed trends closely aligned to those found for the RNA-seq data (Figures 6–8). In most cases, the RT-qPCR relative expression was higher than that found for the RNA-seq data for both up-regulated and down-regulated genes across the various time points examined. These results confirmed and further indicated that genes in *A. thaliana* related to a plant defense response, phytohormone signaling and transduction and systemic acquired resistance (Tables 2–4) were induced by the seaweed extracts and that they may function together against infection by *P. cinnamomi.*

**TABLE 2:** Possible function in defense of individual DEGs significantly induced at least at one time point for each seaweed extract treatment.

Gene	Reported function	References
**AN treatment**		
*PCC1*	Salicylic acid (SA) and Jasmonic acid (JA) triggered pathogen-related response	Mir et al., 2013
*ICS2*	SA biosynthetic process	Shine et al., 2016
*ACD6*	Accelerated cell death 6, activator of the defense response against virulent bacteria and can activate SA-dependent cell death	Lu et al., 2003
*UGT73B3*	Glycosyltransferases, SA induced gene participates in regulation of redox status and general detoxification of ROS−reactive secondary metabolites	Simon et al., 2014
*RLK1*	Receptor like kinase1, pattern recognition receptor which induces innate immune defense	Chaliha et al., 2018
*PDF2.3*	Predicted to encode a PR (pathogenesis-related) protein, belongs to the plant defensin (PDF) family protein, defense response	Sels et al., 2008
*KAT2*	Peroxisomal 3-Ketoacyl-CoA Thiolase 3 (Pkt3), Kat 2, involved in JA biosynthetic process	Pye et al., 2010
*VQ25*	Involved in resistance to necrotrophic pathogen	Cheng et al., 2012
*BAD1*	Ankyrin repeat-containing protein BDA1, involved in plant defense, contribute upstream of NPR1 and WRKY70 to regulate plant defense	Yang et al., 2012
*LURP1*	LURP-one-like protein, required for full basal resistance through R protein to the oomycete pathogen *Hyaloperonospora parasitica*	Knoth and Eulgem, 2008
**DP treatment**		
*GRXC9*	CC-type glutaredoxin protein, involved in SA-dependent disease resistance pathway	Herrera-Vásquez et al., 2015
*TAA1*	Involved in auxin biosynthetic pathway	Stepanova et al., 2008
*CRK5*	Cysteine-rich receptor like protein kinase 5, pathogen-induced *Arabidopsis* gene, involved in multiple distinct defense responses. May function as a disease resistance (R) protein	Chen et al., 2003
*ERF15*	Ethylene responsive factor 15, Transcriptional activator, positively regulates immunity against bacteria and fungi	Zhang et al., 2015
*MYB113*	*MYB113* is critical in the production of anthocyanins which comprise specific stages of phenylpropanoid metabolism	Gonzalez et al., 2009
*IOS1*	Impaired Oomycete Susceptibility 1 (IOS1) has been implicated in defense-related signaling and is important for the resistance against bacteria	Yeh et al., 2016
*MKK9*	Map Kinase Kinase 9, Autophosphorylates and also phosphorylates MPK3 and MPK6. Independently involved in ETH and camalexin biosynthesis. Induces transcription of ACS2, ACS6, ERF1, ERF2, ERF5, ERF6, CYP79B2, CYP79B3, CYP71A13, and PAD3	Xu et al., 2008
*SIB1*	Sigma factor binding protein 1, plays a vital role in JA and SA mediated signaling pathway	Xie et al., 2010
*HR2*	RPW8-like protein 2, contributes to basal resistance to powdery mildew pathogen	Berkey et al., 2017
*PEP1*	Elicitor peptide 1, activates the transcription of plant defense genes and activates the synthesis of hydrogen peroxide	Huffaker et al., 2006
**AN/DP treatment**		
*FMO1*	Flavin-dependent monooxygenase1, involved in critical metabolic SAR signal	Návarová et al., 2012
*EDS16*	Enhanced disease susceptibility 16, involved in SA biosynthesis, Encodes a protein with isochorismate synthase activity. Mutants fail to accumulate salicylic acid. Its function may be redundant with that of ICS2	Wildermuth et al., 2001
*WRKY40*	Pathogen inducible transcription factor involved in ABA signaling pathway	Chen et al., 2010
*JMT*	JA carboxyl methyltransferase, JA biosynthetic pathway	Seo et al., 2001
*MYB75*	Transcriptional regulator of anthocyanin biosynthesis	Borevitz et al., 2000
*CML24*	CaM (Calmodulin)-like protein, acts to induce downstream NO synthesis as intermediary steps in a pathogen perception signaling cascade, leading to innate immune responses	Ma et al., 2008
*ABR1*	Abscisic acid-responsive 1, involved in cell death and defense signaling	Choi and Hwang, 2011
*WAK1*	Wall-associated kinase 1, Induced by SAR conditions, pathogen and defense related signaling molecules including methyl jasmonate (MeJA) and ethylene (Eth)	Meier et al., 2010
*NHL13*	Non-race-specific disease resistance1/harpin-induced1-like13, required for plant immunity to bacteria	Xin et al., 2015
*PBL20*	Probable serine/threonine-protein kinase PBL20, cytoplasmic receptor-like protein kinases, may be involved in plant defense signaling	Zhang et al., 2010

#### Key Plant Defense-Related Genes Significantly Up-Regulated in at Least One Time Point

The most important genes that are related to plant defense that were found to be significantly up-regulated in expression are shown in Tables 2–4. For the AN seaweed extract treatment the analysis of key defense-related genes revealed the presence of SA biosynthetic process and signaling associated genes, JA biosynthetic process and signaling-associated genes, pattern recognition receptors, plant defensin family gene and resistance-related gene active against oomycetes (Tables 2, 3). From an analysis of the DP seaweed extract treatment key defense-related genes revealed were those for SA and JA signaling, R protein encoded, auxin biosynthetic process-associated, phytoalexin production regulating, SAR regulating, basal resistance-related and hydrogen peroxide production associated genes (Tables 2,3). For the AN/DP seaweed extract treatment key defense-related genes found were for SA and JA biosynthetic process-associated genes, ABA-signaling genes, receptor-like protein kinases, an innate immune response inducer gene, SAR-inducible gene, a transcriptional regulator gene and ethylene signaling gene (Tables 2,3). In addition to those sets of genes specific to individual extract treatments there were those that were commonly found across the treatments. Noticeably the three key SAR-associated genes (*PR1*, *PR5*, and *NPR1*) were all up-regulated at 12 hpi following the AN and DP treatments showing a clear involvement of a SA-stimulated pathway. However, for the combined extract treatment at 12 hpi only *NPR1* was up-regulated. In addition, the expression of auxin transporter, hydrogen peroxide responsive, receptor-like protein kinase and secondary metabolite biosynthetic genes were commonly found to be up-regulated across all three seaweed extract treatments (Table 4).

**TABLE 3:** Possible function in defense of individual DEGs significantly induced at least at one time point of at least at two treatments.

Gene	Reported function	References
**AN and DP treatment**		
*BGL2*	beta 1,3-glucanase, involved in systemic acquired resistance	Durrant and Dong, 2004
*AED1*	Apoplastic enhanced disease susceptibility-dependent 1, predicted aspartyl proteases, induced locally and systemically by infection and locally by SA	Breitenbach et al., 2014
*SNC4*	Suppressor of *NPR1-1*, constitutive4 (SNC4) encodes an atypical RLK, involved in plant innate immunity	Bi et al., 2010
**DP and AN/DP treatment**		
*ALD1*	Lys aminotransferase AGD2-like defense response protein 1, required for SAR activation	Song et al., 2004
*BON1*	BONZAI1, is a regulator of defense responses apparently through repressing activity of an R gene	Yang et al., 2006
**AN and AN/DP treatment**		
*AOC3*	Allene oxidase cyclase 3, Key gene in JA biosynthesis	Najafi et al., 2020
*OPR3*	12-oxophytodienoate reductase 2- an isoenzyme involved in JA biosynthesis	Schaller et al., 2000

**TABLE 4:** Commonly up-regulated (at least at one time point) candidate genes following treatment with the three seaweed extracts.

Gene	Reported function
*NPR1*	SA mediated SAR signaling pathway (Eshraghi et al., 2011).
*PR1*	SAR marker gene (Eshraghi et al., 2011).
*PR5*	SAR marker gene (Eshraghi et al., 2011).
*PIN2*	PIN formed protein, Auxin transporter, plays a critical role in auxin gradient−mediated developmental processes, including lateral root formation and gravitropic growth (Sun et al., 2011).
*GR1*	Glutathione reductase 1, plays a crucial role in responses to intracellular hydrogen peroxide and in ensuring appropriate gene expression through both salicylic acid and jasmonic acid signaling pathways (Mhamdi et al., 2010).
*UGT73B4*	UDP-glycosyltransferase 73B4, UGT plays an essential role in the biosynthesis of secondary metabolites in plants (Guo et al., 2016).
*CRK15*	Cysteine-rich receptor like protein kinase 15, involved in pathogen induced plant cell death in *bak1*/*serk4* mutant through regulation of ER quality control (ERQC) (de Oliveira et al., 2016).
MLO-8	Mildew resistance locus O-8, May be involved in modulation of pathogen defense and leaf cell death. Activity seems to be regulated by Ca^2+^-dependent calmodulin binding and seems not to require heterotrimeric G proteins (Devoto et al., 2003).

### Detection of Hydrogen Peroxide in *A. thaliana* Roots

**FIGURE 10:**
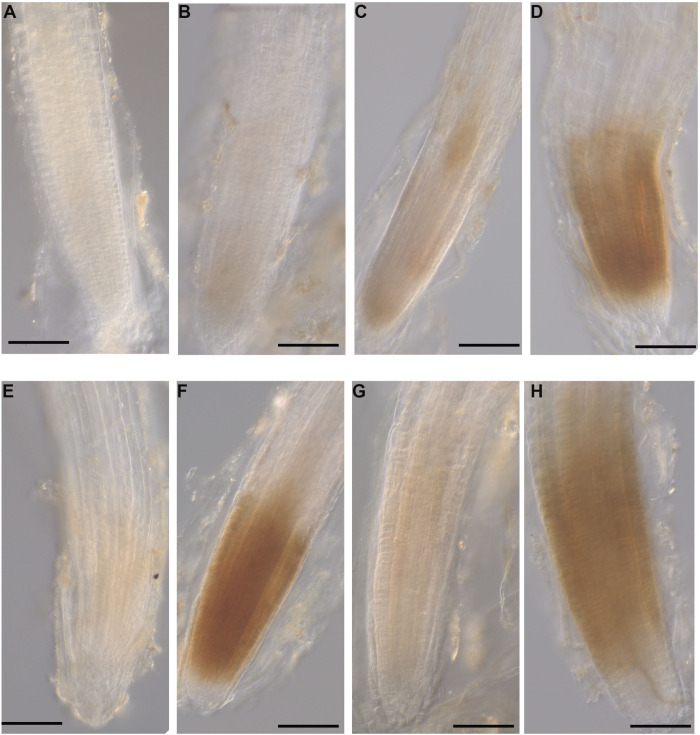
Hydrogen peroxide detection in *A. thaliana* roots grown with seaweed extracts or water as the control and inoculated with *P. cinnamomi* or mock-inoculated with water. Hydrogen peroxide was detected using the 3,3 O-diaminobenzidine tetrachloride (DAB) stain, which resulted in a reddish-brown precipitate in the root tissue. **(A)** Control root grown with water and mock inoculated with water showing no H_2_O_2_ production. **(B)** Control root grown with water and inoculated with the pathogen showing no H_2_O_2_ production. **(C)** Infected root grown with seaweed extract AN and mock inoculated with water showing a low level of H_2_O_2_ production. **(D)** Infected root grown with seaweed extract AN and then inoculated with the pathogen showing H_2_O_2_ production. **(E)** Control root grown with seaweed extract DP and mock inoculated with water showing low level H_2_O_2_ production. **(F)** Infected root grown with seaweed extract DP and inoculated with the pathogen showing H_2_O_2_ production. **(G)** Control root grown with seaweed extract AN/DP and mock inoculated with water showing low level of H_2_O_2_ production. **(H)** Infected root grown with seaweed extract AN/DP and inoculated with the pathogen showing H_2_O_2_ production. Scale bar = 20 μM. Each image is representative of three biological replicates.

The production of H_2_O_2_ was identified as a reddish-brown precipitate that resulted from DAB staining of the *A. thaliana* roots. At 12 hpi, H_2_O_2_ was detected in the roots grown with all three seaweed extracts and inoculated with the pathogen (Figures 10D,F,H). No H_2_O_2_ was detected in control roots grown with water or mock-inoculated with water (Figure 10A). Moreover, a minimal level of H_2_O_2_ was found in all three extract-treated and mock inoculated roots (Figures 10C,E,G). In addition, image analysis of DAB stained roots showed significantly higher stain in each seaweed extract-treated and inoculated root compared to either non-inoculated of each seaweed extract-treated root or water control (Supplementary Figure 15).

## Discussion

### Verification of the *Arabidopsis thaliana-Phytophthora cinnamomi* Plant-Pathogen System

This study used the model plant *A. thaliana* and the generalist, globally devastating pathogen *P. cinnamomi*, to examine the impacts of treatment of plants with two selected brown algal extract-based biostimulants or their combination, on pathogen growth and development in roots. Several previous studies have shown that applications of various brown algal extracts, either to soil or to foliage, enhanced root growth and plant development (Arioli et al., 2015; Mattner et al., 2018). Here we have used a sand culture system to grow *A. thaliana* with extracts from *A. nodosum* (“AN”), or *D. potatorum* (“DP”), or their combination (“AN/DP”). Studies on biostimulants and their impacts on plant disease establishment and progress have been reported (see for example, Gunupuru et al., 2019) although a comprehensive time course study of a root pathogen, and in this case an oomycete root pathogen, in the model plant *A. thaliana* has not been undertaken. The advantage of using *A. thaliana*, apart from its incredibly well detailed and characterized genome, is that there is a growing body of information around the interaction of this host with a range of oomycete pathogens including *P. cinnamomi* (Robinson and Cahill, 2003; Rookes et al., 2008), *Phytophthora porri* (Roetschi et al., 2001) and *P. parasitica* (Le Berre et al., 2017), *Hyaloperonospora arabidopsidis* (Kunz et al., 2008; Ried et al., 2019), and *Albugo candida* (Cooper et al., 2008), but none on their interactions with biostimulants.

In other host−pathogen systems treatment with seaweed extract-based biostimulants have indicated that disease incidence and severity may be reduced following infection. For example, commercial seaweed extracts from *A. nodosum* and *D. potatorum* were found to suppress disease caused by *Plasmodiophora brassicae* in broccoli (Wite et al., 2015) and an extract from *A. nodosum* reduced the severity of *Fusarium* head blight caused by *F. graminearum* in wheat (Gunupuru et al., 2019). It is worth noting the diversity in seaweed extracts. Liquid seaweed extracts are processed from seaweed biomass using different chemical approaches (such as acid and alkaline extraction) and cellular disruption under pressure (Arioli et al., 2015). The extracts comprise diverse molecules that are heterogenous in nature and representative of the extraction process, which emphasizes the need to characterize their properties. The results of our study show that the extent of colonization by *P. cinnamomi* of roots of *A. thaliana* was suppressed by pre-treatment of roots with the alkaline-based extracts from both *A. nodosum* and *D. potatorum* and a mixture of both. The generalist pathogen, *P. cinnamomi*, is an aggressive pathogen that is able to infect close to 5000 plant species (Hardham and Blackman, 2018). Therefore, the suppression of this pathogen by seaweed extracts is a significant finding that demanded further investigation of the details of the potential resistance mechanisms stimulated by different seaweed extracts against *P. cinnamomi* infection.

The availability of genetic and genomic tools for the model plant *A. thaliana* makes it a very good system in which to investigate the *in planta* action of seaweed extracts. Infections by *Phytophthora* spp. in *A. thaliana* have not been found under natural conditions, but have been achieved for several *Phytophthora* species under laboratory conditions (Herlihy et al., 2019). Ecotypic variation to infection by *P. cinnamomi* was described in an earlier study where ecotype Ler was found to be moderately susceptible (Robinson and Cahill, 2003). The microscopic analysis performed in the present study has confirmed that the system that was established and optimized was one in which the plants were successfully inoculated and that the pathogen grew both on the root surface and within roots.

### Effect of Seaweed Extracts on Key Regulatory Resistance-Related Genes

Various seaweeds are a rich source of unique bioactive compounds, for example fucans, carrageenans, ulvans, and laminarins that have been shown to induce plant defense against a variety of pathogens (Cluzet et al., 2004; Shukla et al., 2016, 2019). These elicitor-like molecules may act as priming molecules or pathogen-associated molecular patterns (PAMPs) and thereby activate induced systemic resistance (ISR) and SAR responses. To first test this hypothesis in our system, we examined the expression of three SA- and two JA/ET-responsive marker genes (*PR1*, *NPR1, PR5* and *PDF1.2, THI2.1*, respectively) that are related to SAR (Eshraghi et al., 2011). In our study, each seaweed extract was found to enhance the expression of the key SA-marker genes from the earliest time point tested after inoculation following treatment with seaweed extracts. The JA-marker genes showed variation in expression depending on extract type and time after inoculation. Moreover, the genes were not induced when the plants were treated with the seaweed extracts alone. Other studies have shown similar upregulation of these genes at a single time point. For example, it had been shown earlier that the expression of the *PR1* gene in *A. thaliana* was up-regulated at 24 h post-treatment with an *A. nodosum*-based extract (Cook et al., 2018; Shukla et al., 2019). Furthermore, carrot plants primed by *A. nodosum*-derived extracts induced the accumulation of transcripts of the same or similar genes (Jayaraj et al., 2008). In contrast to these limited studies, the current study has identified the induction of key regulatory genes across a range of time points after pathogen infection therefore providing a post-infection, spatio-temporal analysis of induction following various seaweed extract treatments.

### Transcriptome Analysis Revealed the Complexity of Resistance Induced by Seaweed Extracts

#### Summary of Transcriptional Changes Induced by Each Seaweed Extract With or Without Root Infection by *P. cinnamomi*

Transcriptome analysis using RNA-seq was performed to explore the whole plant transcriptome to reveal correlations between seaweed extract treatment and pathogen suppression that was found following quantitative analysis of the amount of pathogen within roots. Overall the results show that there was a large number of genes that were either up-regulated or down-regulated following exposure of plant roots to each of the extracts. In this study we have specifically concentrated on genes that were up-regulated in these interactions. Three major SAR-related genes were found to be up-regulated in common between extracts and were confirmed by both RNA-Seq and qRT-PCR validation. Equally importantly, each seaweed extract was found to exert its effect through different subsets of genes. A number of studies explored plant transcriptomes following abiotic and biotic stress (see for example: Tommasini et al., 2008; Allardyce et al., 2013; GonñI et al., 2016; Islam et al., 2018; Jithesh et al., 2019), however, the present study is the first report of a comprehensive transcriptome analysis, following treatment of plants with seaweed extracts, root pathogen infection and analysis over multiple time points.

The up-regulated genes were broadly identified as being involved in phytohormone signaling, defense responses, hydrolase activity and the biosynthesis of antibiotics, and also transcription factors and transcription regulators that were involved in metabolite biosynthesis. The GSEA using both Reactome and MapMan databases indicated that the DEGs were involved in a diverse range of activities during seaweed extract-induced plant defenses. For example, brassinosteroid (BR) signaling was commonly found across all three treatments. BRs are plant steroidal hormones that play vital roles in not only plant growth and development but also in plant defense through coordination with other phytohormones (Saini et al., 2015). Another example, proteases (serine or aspartic proteases), were commonly enriched in both AN and DP treatments. Plant genomes encode a large number of proteases which play a regulatory role in a number of processes that are essential for immune responses, more specifically, programmed cell death (PCD) (Balakireva and Zamyatnin, 2018). Most importantly, an array of proteolysis-related genes and their increased expression was commonly found at all time points for each seaweed extract treatment. Proteolysis machinery acts mainly in a housekeeping role to remove non-functional proteins, however, proteolysis has also been shown to play a key role in the recognition of pathogens and the subsequently induced effective defense responses (van der Hoorn and Jones, 2004). Therefore, the results of our study indicated the deployment of multiple phytohormones and proteolytic machinery in seaweed extract-induced defense against *P. cinnamomi*. However, there is considerable scope to further investigate the role of individual proteases in seaweed extract-induced defenses.

The plant cell wall is a dynamic and highly controlled structure that is essential for growth and development. It is considered to be a passive defense barrier against a variety of attackers. Plants have mechanisms that maintain cell wall integrity which comprise a set of so-called “plasma membrane-resident sensors” and “pattern recognition receptors” (Bacete et al., 2018; De Lorenzo et al., 2019). When a pathogen alters the cell wall integrity during epidermal penetration or through deeper colonization of sub-epidermal cells, plants activate suites of genes for cell wall biosynthesis and remodeling as repair and defense responses. This activation of genes and the production of their downstream products is very effective at stopping, or slowing down, pathogen ingress. Several studies, including those that have used overexpressor mutants, have demonstrated the central importance of cell wall-related genes in enabling increased disease resistance (Miedes et al., 2014; Bacete et al., 2018). In our study, cell-wall associated genes were dominant at all-time points following infection for each seaweed extract treatment.

Cell walls are the first line of defense and their modification a very early response to pathogen attack. Each seaweed extract stimulated cell wall-related gene activity following pathogen attack that was well above that for the water-treated control. The induction of these genes at early stages of infection for each seaweed extract treatment was strongly indicative of their contribution toward strengthening the cell wall against pathogen penetration. The induction of similar genes at later stages suggested their contribution to cell wall repair and the fortification of new cell walls. For example, the *MYB46* transcription factor that was up-regulated at 12 hpi in the AN treatment, is directly involved in regulation of the expression of genes responsible for secondary cell wall formation including lignin and cellulose biosynthesis (Miedes et al., 2014). Another example, *CALS5* (Callose synthase 5) that was up-regulated at 6 hpi in the AN/DP treatment, is involved in callose synthesis and was also a pathogen-induced gene in *A. thaliana* infected with the downy mildew pathogen *Hyaloperonospora arabidopsis* (Dong et al., 2008). Callose is a well-known plant defense component and is considered an effective barrier against pathogen invasion including in various *A. thaliana* ecotypes infected by *P. cinnamomi* (Robinson and Cahill, 2003).

The baseline of plant defense is the activation of PRRs localized in the plasma membrane upon recognition of PAMPs/MAMPs (Bigeard et al., 2015). Indeed the induction of *RLK1* in AD-treated plants indicated the activation of PAMP-triggered immunity in these plants against the pathogen. The plant hormones SA, JA and ET have a significant role in plant defense against pathogens. The SA signaling pathway that activates programmed cell death is effective against biotrophic pathogens whereas JA and ET signaling pathways are effective against necrotrophic pathogens (Glazebrook, 2005). The upregulation of both JA and SA biosynthetic or signaling genes suggested the activation by seaweed extracts of both pathways in response to *P. cinnamomi*. The phytohormone, auxin, is well known to be a regulator of plant growth and development. However, auxin is also being recognized as a key regulator of plant defense (Wang and Fu, 2011; Wang et al., 2011). In our study, for example, the upregulation of *TAA1*, a gene involved in auxin biosynthesis, in DP-treated plants indicated the involvement of auxin signaling pathways in response to the pathogen. Similarly, ABA is mainly involved in abiotic stress tolerance as well as in biotic stress but it also may promote plant defense in a complicated network of synergistic and antagonistic interactions (Ton et al., 2009). The induction of an ABA biosynthesis-related transcription factor gene (*WRKY40*) and ABA responsive gene (*ABR1*) in plants treated with AN/DP, along with other key phytohormone-related genes suggested the activation of multiple phytohormone signaling pathways following seaweed extract treatment.

The second layer of plant defense is based on plant disease resistance, (R) gene, mediated resistance by recognition of the products of pathogen avirulence genes and subsequent effector-triggered immunity (ETI) (Andersen et al., 2018). For example, in the current study the induction of *CRK5*, which likely functions as a receptor-like kinase (Chen et al., 2003), in DP-treated plants indicated that ETI may have been triggered. WRKY transcription factors are encoded by a large gene superfamily with a broad range of roles in plants and several groups have reported that proteins containing a short VQ motif interact with WRKY motifs. One of these, VQ25, was reported by Cheng et al. (2012), to be involved in resistance to the necrotrophic pathogen *Botrytis cinerea*. The induction of the candidate resistance-related gene *VQ25* in plants treated with AN thus indicated a contribution of this gene to AN-induced plant defense.

The recent review published by Shukla et al. (2019) presented additional information on some of the plant defense components activated by different extracts from *A. nodosum*. The bioactive compounds present in the prepared *A. nodosum* alkali extract (ANE) were proposed to elicit defense responses to pathogens. The application of ANE enhanced the activation of various enzymes including peroxidases and phenylalanine ammonia-lyase. In addition, ANE also induced ISR against *P. capsici*, another oomycete pathogen, that caused disease in tomato. Further, ANE induced SA-related genes and several JA-related genes such as *PDF1.2* and plant immune response genes such as *WRKY30* and *CYP71A12*, that we also highlight in our study. The review emphasized the information gap around the role of phytohormones in activating defense-related genes that we have now gone some way to fill. For example, the up-regulation of candidate genes, such as *PCC1*, *ACD6*, *GR1*, *ERF014*, *AOC3*, *ACS9*, and *ACS11* all hormone-related by the different extracts derived from both *A. nodosum* and *D. potatorum* and their combination used in our study.

The array of plant defense responses that are activated during pathogen invasion requires an abundant supply of energy which is predominantly derived from primary metabolic processes. These primary metabolic pathways are used by plants not only as a source of energy to drive diverse defense responses, but also as a source of signaling molecules to directly or indirectly, trigger defense responses (Rojas et al., 2014). In the current study primary metabolic pathway activation following pathogen infection was a key outcome of seaweed extract treatment and presumably acted as an energy provider and regulator of *Arabidopsis* defense responses. For example, purine metabolites provide an ongoing source of nitrogen for *A. thaliana* growth. One of the purine metabolites, allantoin, plays a role in a JA-signaling pathway, suggesting that the role of purine metabolism not only underpins normal plant growth but, as others have found, is also a player in stress hormone homeostasis and signaling (Takagi et al., 2016). In our study, a large number of DEGs from each treatment were classified into purine metabolism through KEGG analysis. Therefore, this result indicated that purine metabolites acted to maintain plant growth during pathogen infection as well as contributing to defense-related hormone signaling pathways. In addition, at 12hpi the highest number of up-regulated purine metabolism genes was found for both AN and DP treatments whereas it was only at 24 hpi for the AN/DP treatment. This difference may have indicated a more sustained defense activation and supply of energy in the AN/DP-treated plants during pathogen infection.

Thiamine metabolism has an important function in many metabolic reactions including in glycolysis, the pentose phosphate pathway and the tricarboxylic acid cycle. In addition, thiamine is also related to the induction of SAR and is involved in plant adaptation toward biotic and abiotic stresses (Kamarudin et al., 2017). For example, several studies reported that thiamine treatment of plants, including *A. thaliana*, activated plant defense and enhanced resistance to disease (Ahn et al., 2007; Boubakri et al., 2012; Kamarudin et al., 2017). Therefore, up-regulation of thiamine metabolism which was demonstrated in our study has strong implications for its involvement in the induction of defense, as well as adaptation, during infection by *P. cinnamomi*.

#### Common Plant Defense-Related DEGs That Were Up-Regulated Following Inoculation With *P. cinnamomi* in Extract-Treated Plants

Two hundred and sixty three genes (1.3% of the genome) were commonly found to be up-regulated for at least one time point following inoculation with *P. cinnamomi* of extract-treated plants. A number of candidate resistance-related genes were found to be up-regulated across all treatments including *PIN2*, *GRI*, *UGT73B4*, *CRK15*, and *MLO-8* which have been implicated in diverse resistance-related roles in different host and pathogen combinations. In addition to these genes, even though not above the cutoff by our RNASeq analysis, the pathogenesis-related genes *PR1*, *NPR1*, and *PR5* were confirmed to be commonly up-regulated following treatment with the extracts through our preliminary semi-quantitative PCR analysis as well as in the RNA-seq validation that used quantitative PCR. The PR proteins are a group of proteins that are induced by phytopathogens through activation of specific defense-signaling pathways and are fundamental components of resistance regulation (Backer et al., 2019). After pathogen infection, activation of defense-signaling pathways, such as those regulated by SA and JA take place which further leads to the accumulation of PR proteins that stops pathogen growth and development within host tissues. The SA pathway is especially active following infection by biotrophic pathogens and which stimulates the transcription of *NPR1* which in turn leads to activation, as well as accumulation, of SA-induced *PR* signature gene (*PR1*, *PR2*, and *PR5*) products locally and systemically that leads to SAR (Ali et al., 2018; Backer et al., 2019). PIN proteins are responsible for polar localization in the plasma membrane that determines the direction and rate of intercellular auxin flow (Sun et al., 2011). Moreover, *GR1* plays a crucial role in coordinating gene expression through both SA- and JA-signaling pathways (Mhamdi et al., 2010). The induction of these genes and other phytohormone-related genes in our study suggested that all three extracts induced defense against *P. cinnamomi* that was dependent on the activation of multiple phytohormone signaling pathways. In addition to all the above defense interactors, receptor-like kinases such as *CRK15* found to be up-regulated across treatments in our study, are fundamental signaling components that regulate a variety of cellular processes (Lee et al., 2017).

Plant secondary metabolites have numerous functions in plant−pathogen interactions and experimental evidence has demonstrated their important contributions in plant innate immunity (Piasecka et al., 2015). Plant-produced antibiotics are antimicrobial secondary metabolites and can be broadly classified as phytoalexins and phytoanticipins (Morrissey and Osbourn, 1999). UGT for example, plays an essential role in the biosynthesis of secondary metabolites in plants (Guo et al., 2016) and the induction of *UGT73B4* and other genes associated with the biosynthesis of antibiotics in all seaweed extract treatments indicated the synthesis of potentially novel antimicrobial compounds as a reaction to infection by *P. cinnamomi.*

The diverse patterns of differential gene expression found in our study were consistent with seaweed extracts having complex and pleiotropic modes of action that involved a cascade of gene activation for different plant responses. The commonality in the transcriptome profiles suggested that, at least for the seaweed extracts derived from the brown seaweeds used in the current study, behaved in a similar, but not identical, way.

#### Novel Genes That Were Up-Regulated That Provide Insight Into the Mechanisms of Action of Seaweed Extracts Against *P. cinnamomi*

WRKY transcription factors play important roles in plant responses to various biotic and abiotic stresses. WRKYs act as substrates of calcium-dependent protein kinases and calmodulin (CaM) is a Ca^2+^ -binding protein that is involved in various cellular functions (Bakshi and Oelmüller, 2014). The function of calmodulin-like (CML) proteins is largely unknown. However, one of these, CML8 was found to be up-regulated in our study and has been shown to be involved in *Arabidopsis* immunity against *Pseudomonas syringae* (Zhu et al., 2017). The strong and consistent upregulation of *WRKY42* and *CML8* in the AN treatment indicated a correlation with, and the increased involvement of, calcium signaling in defense activation.

The *A. thaliana* genome has four jasmonate-induced oxygenases (JOXs) and one of them hydroxylates jasmonic acid to 12-OH-JA (Caarls et al., 2017). In our study the higher expression of *JOX1* in the DP treatment at 6 hpi indicated the involvement of other phytohormone signaling pathways at this stage of the interaction with the pathogen. The expression of the *POLARIS* (*PLS*) gene that encodes a 36-amino acid peptide that regulates plant root growth and vascular development, is related to auxin transport and coordinates the ethylene signaling pathway (Chilley et al., 2006). The strong up-regulation of *PLS* expression in AN/DP treatments suggested that *PLS* also contributed to *A. thaliana* root growth, as well as functioning in regulation of phytohormone-induced signaling pathways, that resulted in suppression of *P. cinnamomi*. In addition, the strong up-regulation of other uncharacterized genes, in all three treatments, suggested the contribution of unknown novel mechanisms in AN and DP extract-induced defense.

Other publications have compared the transcriptional profiles of plants treated with seaweed extracts derived from the brown seaweed *A. nodosum* (Nair et al., 2012; GonñI et al., 2016; Santaniello et al., 2017; Jithesh et al., 2019; Omidbakhshfard et al., 2020). The extracts used in these studies varied in their chemical nature (including alkaline, neutral and acid extracts) and extraction approaches. Despite the differences observed among the transcriptional profiles following extract-treatment of plants, the overall results demonstrate the highly dynamic and responsive nature of plants to different types of seaweed extracts, and the inherent capacity for the seaweed extracts to simultaneously enhance plant growth and tolerance to biotic and abiotic stresses.

### Role of ROS in Seaweed Induced Plant Defense

To examine the production of defense-related components prior to and following the upregulation of defense-related transcripts, the production of hydrogen peroxide (H_2_O_2_), a reactive oxygen species (ROS), was examined in *A. thaliana* roots grown with each seaweed extract and infected, or not, with *P. cinnamomi*. The induction of hydrogen peroxide was found only in those roots treated with the three seaweed extracts individually and infected with *P. cinnamomi*. This result is somewhat different to that of a previous study (Cook et al., 2018) that showed the induction of reactive oxygen species in seedlings treated only with an *A. nodosum* extract. This variation in results between the two studies may reflect differences in preparation of the extract and the treatment and analysis methods. The method used in our study gave a direct visualization of the location and intensity of ROS in the roots, something that is not possible using alternative assays.

The *PEP1* gene, which was found to be up-regulated in our study in DP-treated plants, is involved in activation of the synthesis of enzymes associated with hydrogen peroxide formation (Huffaker et al., 2006). Also, plant peroxidases participate in various physiological processes, such as lignification, suberisation, auxin catabolism and defense mechanisms that are activated during pathogen infection. They are considered to catalyze the generation of aromatic oxyl radicals from several aromatic compounds and the peroxidase-dependent production of such organic radicals often results in the generation of reactive oxygen species (Kagan et al., 1990; Kawano, 2003). The induction of peroxidases in all extract treatments suggested their involvement in ROS generation and potentially other aspects of extract-induced defense mechanisms.

### The Trade-Off Between Growth and Defense and Priming for Defense

New insights into how plants balance growth while responding to stress has implications for advanced agriculture. The compromise between growth and stress response is based on plants having limited resources which need to be prioritized for growth, or toward responses to the abiotic and/or biotic stresses they encounter (Huot et al., 2014; Karasov et al., 2017). The trade-off concept is supported by research that has demonstrated that plant-fitness costs are associated with the induction of defense genes (Huot et al., 2014; Karasov et al., 2017), and that maintaining activated plant response systems is metabolically costly (Karasov et al., 2017; Buswell et al., 2018). Nevertheless, emerging research has uncovered chemical priming of immunity that provides defense without costs to plant growth (Buswell et al., 2018).

In our research we observed a balanced trade-off between root growth and the activation of specific defense pathways. Our transcriptomic analysis identified the up-regulation of specific defense-associated pathways (such as those regulated by SA, JA, ET, and PPP) and genes associated with plant resistance (for example *PR1*, *MLO*, and others), while root length growth continued, despite the interior of the roots being actively infected by *P. cinnamomi*. In our experimental design pretreating the plants with seaweed extract was an important pre-requisite. This approach may have contributed to a favorable trade-off that utilized a natural plant priming system. More generally, extracts of different seaweeds have been shown to activate broad spectrum defense systems in plants (Kerchev et al., 2019; Shukla et al., 2019). Collectively the research supports the notion that seaweed extracts may act as a plant priming stimulant, particularly if pre-applied.

Plant-priming is an adaptive and low-cost defensive mechanism that, upon activation by a priming stimulus, results in a faster and/or stronger induction of inducible defenses. Plant-priming occurs in a wide range of plant species and is often associated with enhanced abiotic and biotic stress tolerance (Martinez-Medina et al., 2016). The idea that priming is the result of treatment with a specific seaweed extract is supported by our transcriptomics analysis particularly based on the molecular and cellular GO categories: for example, genes up-regulated for (i) DNA Binding Transcription Factor Activity (ii) Transcription Regulatory Region DNA Binding, (iii) the increased number of transcripts found in the nucleus, (iv) and up regulation of genes associated with redox signaling and sensing. Also ROS are key molecules involved in the priming process (Borges et al., 2014). Our data showing the accumulation of hydrogen peroxide in root cells at 12 hpi, in plants pretreated with seaweed extract, was consistent with ROS acting as a latent signal involved in priming plant resistance (Gonzalez et al., 2009). The priming of plant resistance can also be achieved by exogenous application of synthetic and natural compounds (Aranega-Bou et al., 2014). Hexanoic acid, for example, is a natural primer (Aranega-Bou et al., 2014) which is a component of one of the seaweed extracts used in this study (AN/DP, data not published).

## Conclusion

This study showed that *A. thaliana* was a very useful model plant for studies on the impact that a seaweed extract-based biostimulant had on interactions at the molecular level with a root pathogen. We also demonstrated the up-regulation of key SAR-related genes and phytohormone- associated genes at various critical time points post-inoculation following treatment with extracts of the selected brown seaweeds. Importantly, each seaweed extract induced multiple defense-related pathways prior to penetration and infection by the pathogen. These observations were characteristic of a primed response, and closely associated with ROS production. Transcriptomic analysis has proven to be a powerful approach to elucidate the timing of activation of defense-related mechanisms and the subsequent suppression of pathogen growth. Our results can now be used in future studies that use specific plant mutants that are impaired in various resistance-related pathways or, for example, gene edited hosts to investigate the role of individual defense components in seaweed extract-induced defense. Further, we propose that the approach used in the current study could be applied to agriculturally important crop species to investigate the impact of seaweed extract-treatment on their reaction to a pathogen.

## Data Availability Statement

The datasets presented in this study have been deposited in online repositories. The names of the repositories and their accession number(s) can be found either in this article or in the/Supplementary Material.

## Author Contributions

MI, DC, and TA conceptualized and designed the project. MI performed the laboratory work, analyzed the RNA-seq transcriptome data, and wrote and revised the manuscript. HG contributed to RNA-seq data processing. MZ performed the functional analysis of DEGs. HH contributed to the critical analysis of the RNA-seq data and manuscript draft. DC and TA contributed to the manuscript draft and final version. All authors revised and approved the final version to be published and agreed with all aspects of the work.

## Conflict of Interest

Seasol^®^ was manufactured by the company Seasol International (SI). TA and HH are researchers in the SI R&D Department and employed by SI. SI provided in-kind support as salary for TA and HH. The remaining authors declare that the research was conducted in the absence of any commercial or financial relationship that could be construed as a potential conflict of interest.

## Footnotes

1 www.arabidopsis.com

2 https://www.ncbi.nlm.nih.gov/sra/PRJNA609590

3 http://www.genome.jp/kegg
