# Supplementary material for: Phaeophyceaean (Brown Algal) Extracts Activate Plant Defense Systems in Arabidopsis thaliana Challenged With Phytophthora cinnamomi
Source: Front Plant Sci. 2020 Jul 7;11:852. doi: 10.3389/fpls.2020.00852 (PMC7381280; doi:10.3389/fpls.2020.00852)
Supplement: Supplementary file 1 [file Table_1.docx]

**Supplementary Table 1.** Primer pair sequences used in semi-quantitative PCR

| **Gene** | **AGI number** | **Forward and reverse primer (5'-3')** | **Amplicon size** | **Source of Primer** |
| --- | --- | --- | --- | --- |
| *PR1* | AT2G14610 | TTCTTCCCTCGAAAGCTCAA  AAGGCCCACCAGAGTGTATG | 174 | This study |
| *PR5* | AT1G75040 | CGTACAGGCTGCAACTTTGA  TGAATTCAGCCAGAGTGACG | 112 | This study |
| *NPR1* | AT1G64280 | CCAAGTGGGACATGGTCAGG  TGCATGATCCATGTTTGGGT | 91 | Eshraghi *et al.* 2011 |
| *PDF1.2* | AT5G44420 | CCAAGTGGGACATGGTCAGG  TGCATGATCCATGTTTGGCT | 91 | Eshraghi *et al.* 2011 |
| *THI2.1* | AT1G72260 | ACGCCATTCTCGAAAACTCAG  TGGAGAGTGTTCATGGCACC | 91 | Eshraghi *et al.* 2011 |
| *EF1-alpha* | AT5G60390 | TGGTGACGCTGGTATGGTTA  TCCTTCTTGTCCACGCTCTT | 147 | This study |
| *Actin* | AT3G18780 | CTTGCACCAAGCAGCATGAA  CCGATCCAGACACTGTACTTCCTT | 68 | Eshraghi *et al.* 2011 |
