# Supplementary material for: Phaeophyceaean (Brown Algal) Extracts Activate Plant Defense Systems in Arabidopsis thaliana Challenged With Phytophthora cinnamomi
Source: Front Plant Sci. 2020 Jul 7;11:852. doi: 10.3389/fpls.2020.00852 (PMC7381280; doi:10.3389/fpls.2020.00852)
Supplement: Supplementary file 2 [file Table_2.docx]

**Supplementary Table 2.** Primer pair sequences used in the RNA-seq validation experiment

| **Gene id (s)** | **Sequence** | **Product length** | **Primer source** |
| --- | --- | --- | --- |
| *BGL2*_For | ATCGCCTAGCATCCCGTAGC | 141 | This study |
| *BGL2*_Rev | ACCACACAGGTTCAGTCATCTT |  |  |
| *PCC1*__For | GTACACAAGTCCGCCACCAA | 93 |  |
| *PCC1*_Rev | ACGCCCTTGGAGTTTGTCTC |  |  |
| *RLK*_For | GCCGTTTGGGGATTATGAGA | 96 |  |
| *RLK*_Rev | CCAAAAATAACCGCAGCACA |  |  |
| *ACD6*_For | CTTCTCGTGGTAGCGGCTCT | 122 |  |
| *ACD6*_Rev | GGGTTAGTGGCCAAAGTTGC |  |  |
| *UGT73B3*_for | CAGGGCTACCAATGGTGACA | 93 |  |
| *UGT73B3*_Rev | CCACGCTCACTCCTGTTCTG |  |  |
| *ACT2*_for | ACCTTGCTGGACGTGACCTT | 88 |  |
| *ACT2*_Rev | ATTTCCCGCTCTGCTGTTGT |  |  |
| *ACT8*_for | AACCAGCTCCTCCATCGAAA | 130 |  |
| *ACT8*_rev | GATCCCTGCAGCTTCCATTC |  |  |
| *TAA1*_for | TGTTGGTTCCTTGAGCCAGA | 103 |  |
| *TAA1*_rev | AACCGGTCCCAACCACTATG |  |  |
| *ALD1*_for | TCGGCTTGGTTGGTCTATCA | 129 |  |
| *ALD1*_rev | AATCCACCAGCCTGAGCAAT |  |  |
| *AED1*_for | ATTATGCTTTGCGTATGTCTCAATTGG | 261 | Breitenbach *et al.*, 2014 |
| *AED1*_rev | GGACTCCACACGTGCTTGATCG |  |  |
| *GRXC9*_for | TCTCAAGGACCGCCGGATTC | 129 | This study |
| *GRXC9*_rev | ACGGCGAGAGAGTTCGGATG |  |  |
| *MYB75*_for | TTCCTGCACCGGTTTAGCCC | 122 |  |
| *MYB75*_rev | TTGGTGTGCATAGATTCTTCCTGA |  |  |
| *EDS16*_for | TTGGCAGGGAGACTTACGAAGG | 91 |  |
| *EDS16*_rev | AATTCGAAGAAATGAAGAGCTTGGA |  |  |
| *WRKY40*_for | ACCCTCGTAAGCATTCATGAAAAT | 128 |  |
| *WRKY40*_rev | CCTCCATTGATACCCATCTTTCACA |  |  |
| *JMT*_for | CCTCGTCCATTACTCTTCTGCC | 146 |  |
| *JMT*_rev | TCGTGTACACATACATACGAGTGA |  |  |
| *PR1*_for | CGTAAGGCCCACCAGAGTGT | 131 |  |
| *PR1*_rev | TCACAACCAGGCACGAGGAG |  |  |
| *NPR1*_for | TACCAGTGAGACGGTCAGGC | 136 |  |
| *NPR1*_rev | TGTGTCGTCCACTGTTTAGTTGC |  |  |
| *PR5*_for | AGCGGCATTGCTGTTATGGC | 134 |  |
| *PR5*_rev | CGGGAAGCACCTGGAGTCAA |  |  |
