# Supplementary material for: Phaeophyceaean (Brown Algal) Extracts Activate Plant Defense Systems in Arabidopsis thaliana Challenged With Phytophthora cinnamomi
Source: Front Plant Sci. 2020 Jul 7;11:852. doi: 10.3389/fpls.2020.00852 (PMC7381280; doi:10.3389/fpls.2020.00852)
Supplement: Supplementary file 3 [file Table_3.docx]

**Supplementary Table 3****.** RNA-Seq statistics for reads mapped to the *A. thaliana* genome

| **Sample id** | **% mapped in pairs** | **% mapped in broken pairs** | **% reads not mapped to genome** |
| --- | --- | --- | --- |
| **Water control** | | | |
| H-0 | 96.99 | 8.80 | 3.04 |
| H-3 | 97.46 | 8.09 | 2.54 |
| H-6 | 93.79 | 7.47 | 3.39 |
| H-12 | 97.04 | 7.72 | 2.96 |
| H-24 | 96.00 | 7.53 | 4.00 |
| **AN treatment** | | | |
| AN-0 | 89.81 | 6.79 | 3.40 |
| AN-3 | 91.31 | 5.92 | 2.77 |
| AN-6 | 93.24 | 4.05 | 2.72 |
| AN-12 | 89.40 | 7.83 | 2.77 |
| AN-24 | 89.70 | 7.07 | 3.23 |
| **DP treatment** | | | |
| DP-0 | 90.02 | 6.7 | 3.28 |
| DP-3 | 90.67 | 6.35 | 2.98 |
| DP-6 | 91.07 | 5.76 | 3.17 |
| DP-12 | 92.43 | 4.92 | 2.65 |
| DP-24 | 91.59 | 5.22 | 3.19 |
| **AN/DP treatment** | | | |
| AN/DP-0 | 91.08 | 5.87 | 3.05 |
| AN/DP -3 | 87.54 | 9.62 | 2.84 |
| AN/DP -6 | 88.93 | 7.77 | 3.30 |
| AN/DP -12 | 90.11 | 8.71 | 8.77 |
| AN/DP -24 | 87.95 | 2.17 | 3.29 |

Data are presented as the average of three biological replicates
