# Supplementary material for: Phaeophyceaean (Brown Algal) Extracts Activate Plant Defense Systems in Arabidopsis thaliana Challenged With Phytophthora cinnamomi
Source: Front Plant Sci. 2020 Jul 7;11:852. doi: 10.3389/fpls.2020.00852 (PMC7381280; doi:10.3389/fpls.2020.00852)
Supplement: Supplementary file 4 [file Table_4.docx]

**Supplementary Table 4.** The number of up-regulated and down-regulated genes in *A. thaliana* grown with seaweed extracts and infected with *P. cinnamomi*

| **Sample Id** | **Up-regulated genes** | **Down-regulated genes** | **Total DEGs** |
| --- | --- | --- | --- |
| **AN treatment** | | | |
| AN-0 | 205 | 287 | 492 |
| AN-3 | 295 | 823 | 1118 |
| AN-6 | 338 | 386 | 724 |
| AN-12 | 882 | 86 | 968 |
| AN-24 | 417 | 189 | 606 |
| **DP treatment** | | | |
| DP-0 | 91 | 899 | 990 |
| DP-3 | 233 | 777 | 1010 |
| DP-6 | 147 | 363 | 510 |
| DP-12 | 563 | 119 | 682 |
| DP-24 | 582 | 316 | 898 |
| **AN/DP treatment** | | | |
| AN/DP-0 | 369 | 748 | 1117 |
| AN/DP-3 | 222 | 1435 | 1657 |
| AN/DP-6 | 135 | 1104 | 1239 |
| AN/DP -12 | 402 | 429 | 831 |
| AN/DP -24 | 802 | 430 | 1232 |
