# Supplementary material for: Phaeophyceaean (Brown Algal) Extracts Activate Plant Defense Systems in Arabidopsis thaliana Challenged With Phytophthora cinnamomi
Source: Front Plant Sci. 2020 Jul 7;11:852. doi: 10.3389/fpls.2020.00852 (PMC7381280; doi:10.3389/fpls.2020.00852)
Supplement: Supplementary file 5 [file Presentation_1.pdf]

Supplementary Method 1

DNA extraction for *P. cinnamomi* quantification

The roots of *A. thaliana* (collected from 10 plants per time point per replicate) were ground to a fine paste in 500 µL of CTAB buffer and subsequently transferred into a 1.5 mL eppendorf tube. The plant extract mixtures were then incubated in a water bath for 15 min at 55°C and the tubes were centrifuged at 12000 × g for 15 min to spin down the cell debris. Then the supernatant was transferred to another clean tube and 200 µL of chloroform:isoamyl alcohol (24:1) was added to the tube. The solution was mixed by inversion and then centrifuging the tubes at 13000 × g for 1 min. Then the upper aqueous phase was transferred to another clean eppendorf tube and subsequently 50 µL of 7.5 M Ammonium Acetate followed by 500 µL of ice-cold absolute ethanol were added to each tube. Then the tubes were inverted slowly several times before being incubated at -20°C to precipitate the DNA. Following precipitation, the DNA was washed two times using ice-cold 70% ethanol by centrifugation at 13000 × g for 1 min. The washed DNA pellet was then left in the fume hood to evaporate the ethanol for 15 min. Then the DNA was resuspended in 50 µL of sterile DNase free water. After resuspension, the DNA solution was incubated at 65°C for 20 min to destroy any DNases and stored at -20°C until further use.

Primers used for the *P. cinnamomi* quantification experiment:

**Primer name**	**Forward and reverse primer (5'-3')**	**Source of primer**
At_Actin	CTTGCACCAAGCAGCATGAA<br>CCGATCCAGACACTGTACTTCCTT	Engelbrecht *et al.* 2013
Pc_LPV3	GTGCAGACT GTCGATGTG<br>GTGCAGACTGTCGAT GTG	Engelbrecht *et al.* 2013
Pc_LPV3N	GTGCAGACTGTCGATGTG<br>GAGGTGAAGGCTGTTGAG	Engelbrecht *et al.* 2013
