# Supplementary material for: Phaeophyceaean (Brown Algal) Extracts Activate Plant Defense Systems in Arabidopsis thaliana Challenged With Phytophthora cinnamomi
Source: Front Plant Sci. 2020 Jul 7;11:852. doi: 10.3389/fpls.2020.00852 (PMC7381280; doi:10.3389/fpls.2020.00852)
Supplement: Supplementary file 6 [file Data_Sheet_1.docx]

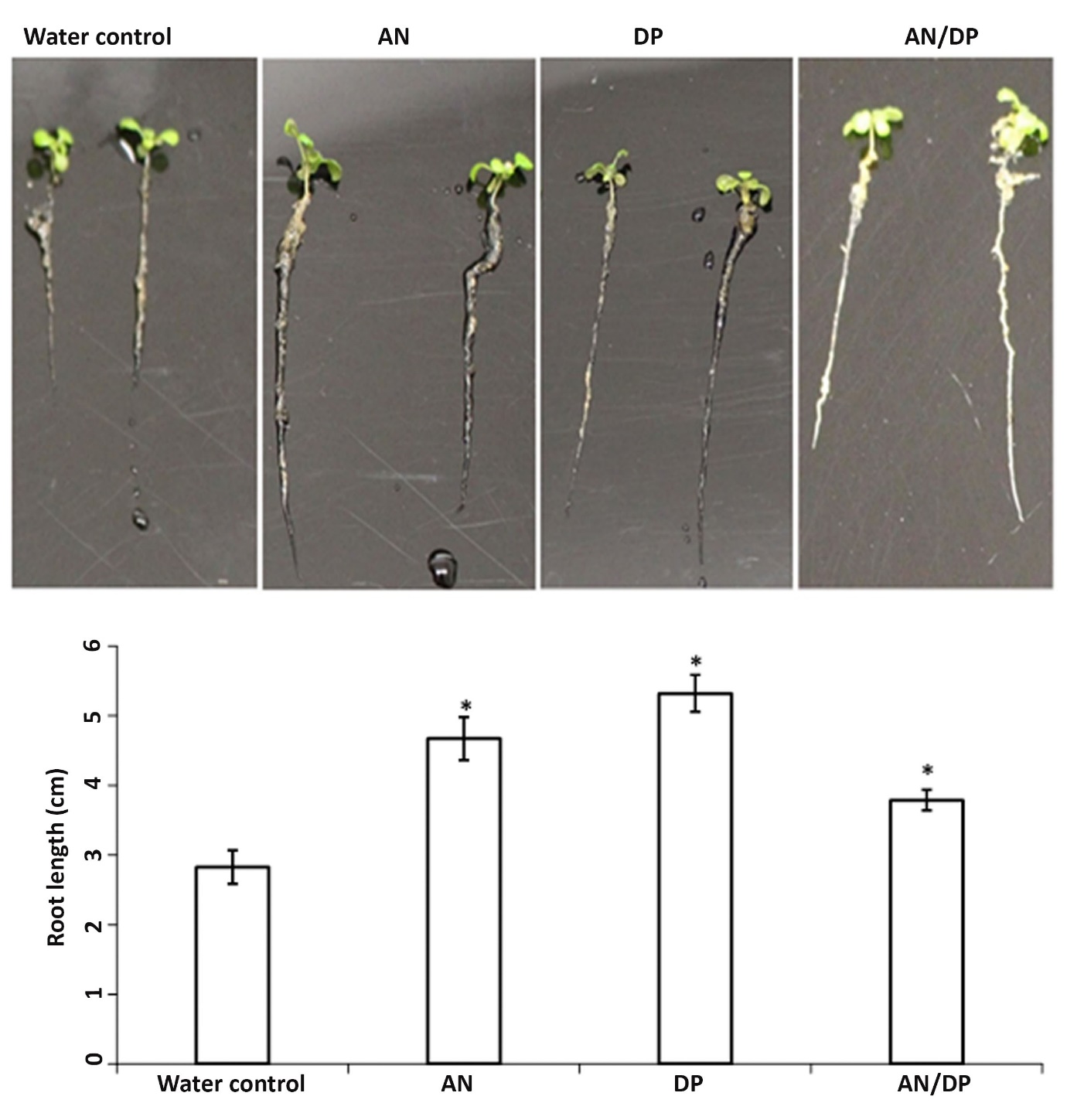


**A**

**B**

**Supplementary Figure 1.** Growth of *A. thaliana* in the sand growth system after 7 days. **A**. Representative images of root growth for seaweed extract-treated plants **B**. Root length of plants treated with either water as the control or seaweed extracts. Error bars represent the standard error of the mean for three biological replicates. DMRT was used to determine the significance of difference between infected and mock control samples. * indicates significant difference at a P value <0.05.
