# Supplementary material for: Phaeophyceaean (Brown Algal) Extracts Activate Plant Defense Systems in Arabidopsis thaliana Challenged With Phytophthora cinnamomi
Source: Front Plant Sci. 2020 Jul 7;11:852. doi: 10.3389/fpls.2020.00852 (PMC7381280; doi:10.3389/fpls.2020.00852)
Supplement: Supplementary file 7 [file Data_Sheet_2.docx]

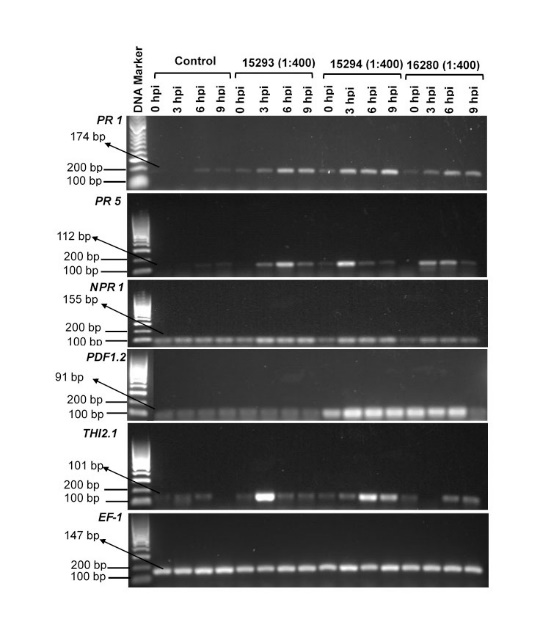


**Water control AN (1:400) DP (1:400) AN/DP (1:400)**

**Supplementary Figure 2.** Agarose gel electrophoresis images of RT-PCR that was performed to analyse the up-regulation of candidate SAR-related genes in *A. thaliana* grown with water control or seaweed extracts (AN, DP, AN/DP) following inoculation with *P. cinnamomi.* The expression levels of *PR1*, *PR5, NPR1, PDF1.2* and *THI2.1* were up-regulated in plants grown with seaweed extracts then inoculated with *P. cinnamomi*. *EF1* was used as an internal control. Images are representative of two independent experiments conducted with two biological replicates.
