# Supplementary material for: Phaeophyceaean (Brown Algal) Extracts Activate Plant Defense Systems in Arabidopsis thaliana Challenged With Phytophthora cinnamomi
Source: Front Plant Sci. 2020 Jul 7;11:852. doi: 10.3389/fpls.2020.00852 (PMC7381280; doi:10.3389/fpls.2020.00852)
Supplement: Supplementary file 8 [file Data_Sheet_3.docx]

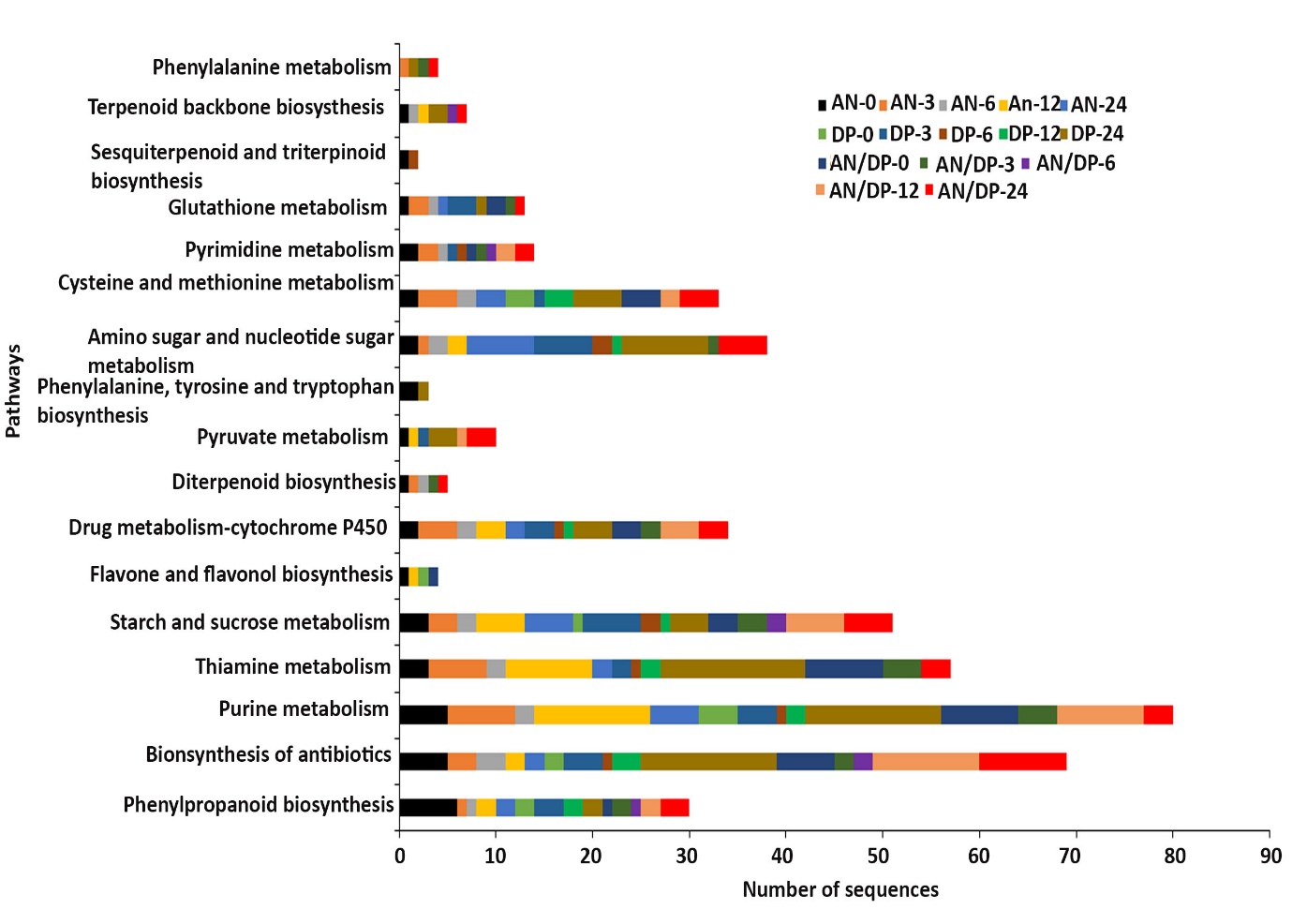


**Supplementary Figure 3**. KEGG pathway analysis (http://www.kegg.jp/kegg/kegg1.html) for DEGs of treatment with three seaweed extracts (AN, DP and AN/DP) at various time point after inoculation with *P. cinnamomi*
