# Supplementary material for: Phaeophyceaean (Brown Algal) Extracts Activate Plant Defense Systems in Arabidopsis thaliana Challenged With Phytophthora cinnamomi
Source: Front Plant Sci. 2020 Jul 7;11:852. doi: 10.3389/fpls.2020.00852 (PMC7381280; doi:10.3389/fpls.2020.00852)
Supplement: Supplementary file 9 [file Data_Sheet_4.docx]

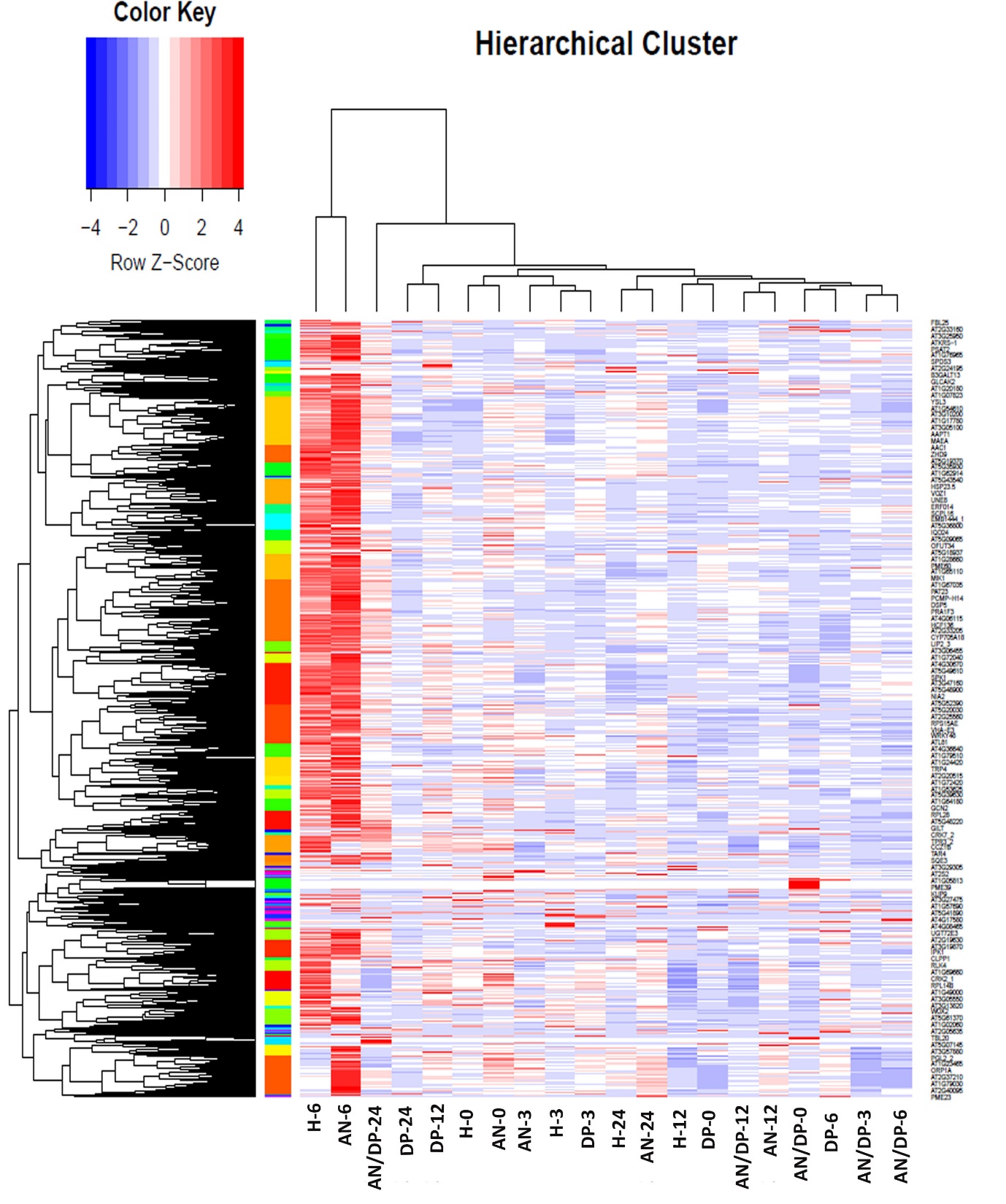


**Supplementary Figure 4:** Hierarchical clustering of DEGs, using the RNA sequencing data derived from 5 time points for each treatment (water as a control-H, AN, DP and AN/DP) based on expression values. The red bands indicate higher expression and the blue bands show lower expression.
