# Supplementary material for: Phaeophyceaean (Brown Algal) Extracts Activate Plant Defense Systems in Arabidopsis thaliana Challenged With Phytophthora cinnamomi
Source: Front Plant Sci. 2020 Jul 7;11:852. doi: 10.3389/fpls.2020.00852 (PMC7381280; doi:10.3389/fpls.2020.00852)
Supplement: Supplementary file 10 [file Data_Sheet_5.docx]

**H-0_vs_AN-0**

**H-3_vs_AN-3**

**H-6_vs_AN-6**

**H-12_vs_AN-12**

**H-24_vs_AN-24**

**Supplementary Figure 5.** Expression patterns of 30 DEGs showing highest fold change for plants treated with AN and then inoculated with *P. cinnamomi*. The Z-score of each gene is presented using a color scale. The right side of each heatmap indicates gene ID of *A. thaliana*.
