# Supplementary material for: Phaeophyceaean (Brown Algal) Extracts Activate Plant Defense Systems in Arabidopsis thaliana Challenged With Phytophthora cinnamomi
Source: Front Plant Sci. 2020 Jul 7;11:852. doi: 10.3389/fpls.2020.00852 (PMC7381280; doi:10.3389/fpls.2020.00852)
Supplement: Supplementary file 11 [file Data_Sheet_6.docx]

**H-0_vs_DP-0**

**H-3_vs_DP-3**

**H-6_vs_DP-6**

**H-12_vs_DP-12**

**H-24_vs_DP-24**

**Supplementary Figure 6.** Expression patterns of 30 DEGs showing highest fold change for plants treated with DP and then inoculated with *P. cinnamomi*. The Z-score of each gene is presented using a color scale. The right side of each heatmap indicates gene ID of *A. thaliana*.

.
