# Supplementary material for: Phaeophyceaean (Brown Algal) Extracts Activate Plant Defense Systems in Arabidopsis thaliana Challenged With Phytophthora cinnamomi
Source: Front Plant Sci. 2020 Jul 7;11:852. doi: 10.3389/fpls.2020.00852 (PMC7381280; doi:10.3389/fpls.2020.00852)
Supplement: Supplementary file 12 [file Data_Sheet_7.docx]

**H-0_vs_AN/DP-0**

**H-3_vs_ AN/DP -3**

**H-6_vs_ AN/DP -6**

**H-12_vs_ AN/DP -12**

**H-24_vs_ AN/DP -24**

**Supplementary Figure 7.** Expression patterns of 30 DEGs showing highest fold change for plants treated with AN/DP and then inoculated with *P. cinnamomi*. The Z-score of each gene is presented using a color scale. The right side of each heatmap indicates gene ID of *A. thaliana*.
