# Supplementary material for: Phaeophyceaean (Brown Algal) Extracts Activate Plant Defense Systems in Arabidopsis thaliana Challenged With Phytophthora cinnamomi
Source: Front Plant Sci. 2020 Jul 7;11:852. doi: 10.3389/fpls.2020.00852 (PMC7381280; doi:10.3389/fpls.2020.00852)
Supplement: Supplementary file 15 [file Data_Sheet_10.docx]

**
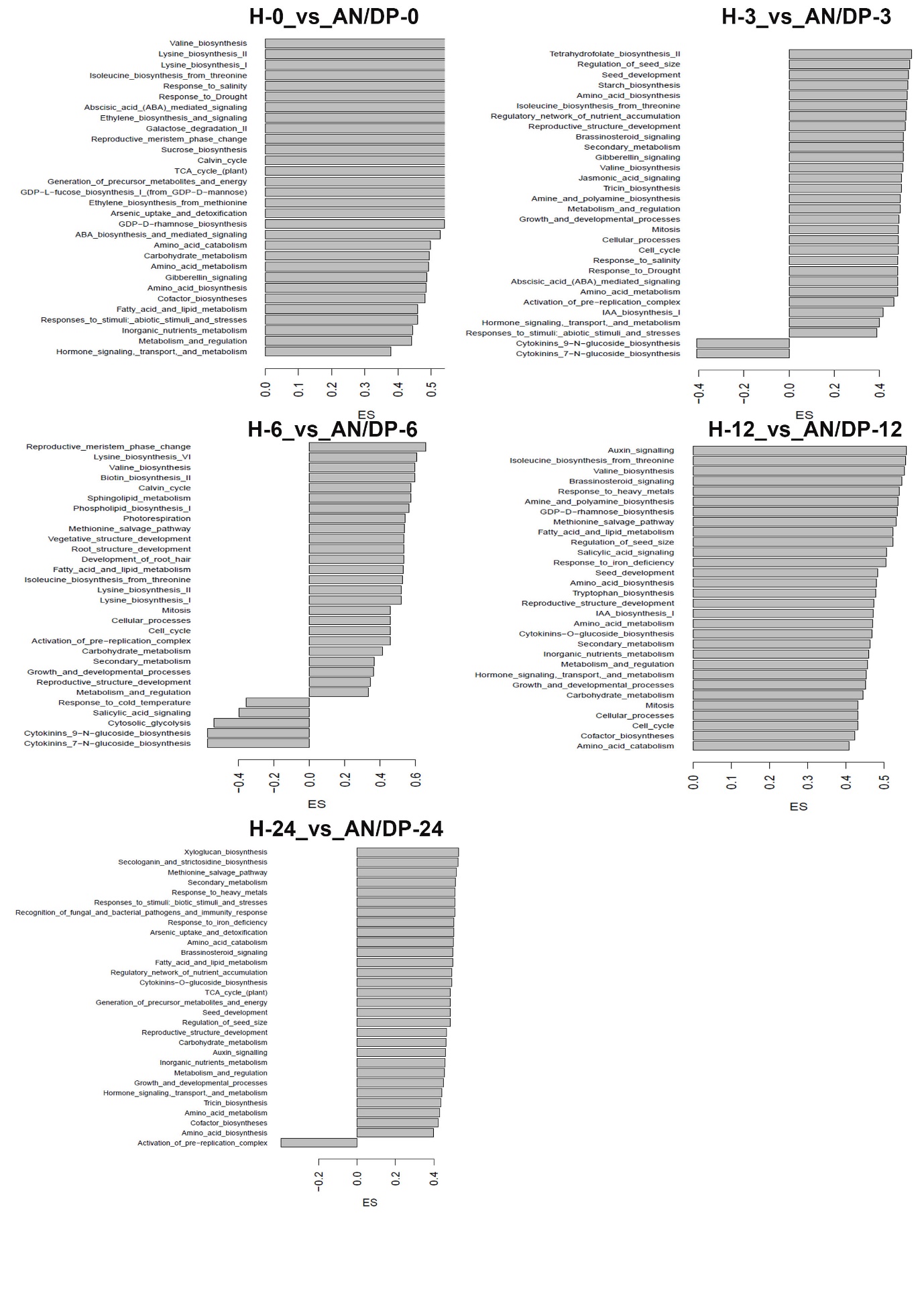
Supplementary Figure 10.** Overview of enriched functional groups extracted from GSEA using the Reactome database for DEGs following treatment of plants with AN/DP and at different time points after inoculation with *P. cinnamomi.* ES= Enrichment score.
