# Supplementary material for: Phaeophyceaean (Brown Algal) Extracts Activate Plant Defense Systems in Arabidopsis thaliana Challenged With Phytophthora cinnamomi
Source: Front Plant Sci. 2020 Jul 7;11:852. doi: 10.3389/fpls.2020.00852 (PMC7381280; doi:10.3389/fpls.2020.00852)
Supplement: Supplementary file 19 [file Data_Sheet_14.docx]

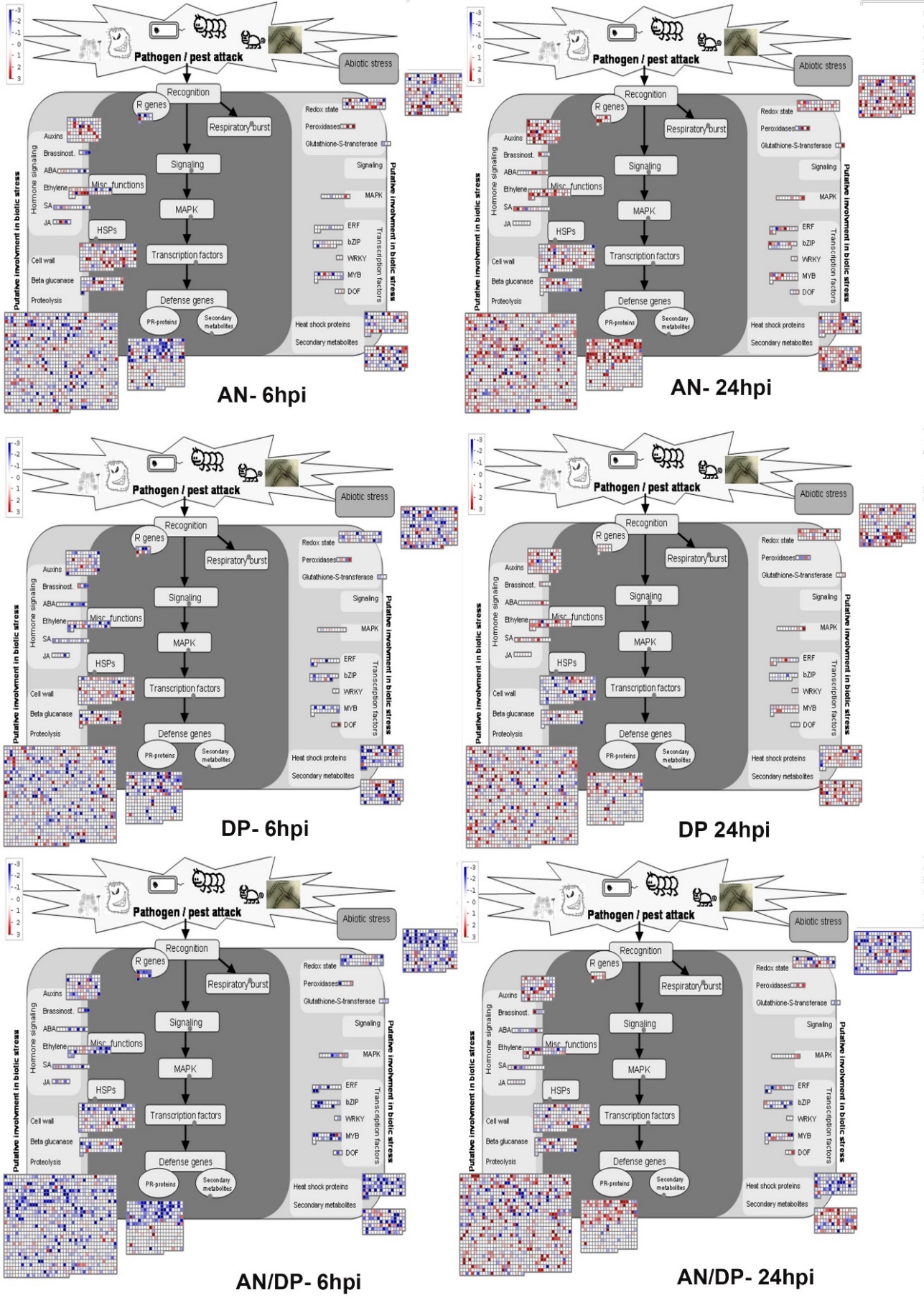


**Supplementary Figure 14.** MapMan overview of DEGs affected by each seaweed extract treatment (AN, DP and AN/DP) followed by infection with *P. cinnamomi* (at 6 and 24 hpi) related to biotic stress. Average fold change of gene expression is indicated by the colour scale (red represents up-regulated gene expression and blue represents down-regulated gene expression)
