# Supplementary material for: Phaeophyceaean (Brown Algal) Extracts Activate Plant Defense Systems in Arabidopsis thaliana Challenged With Phytophthora cinnamomi
Source: Front Plant Sci. 2020 Jul 7;11:852. doi: 10.3389/fpls.2020.00852 (PMC7381280; doi:10.3389/fpls.2020.00852)
Supplement: Supplementary file 20 [file Data_Sheet_15.docx]

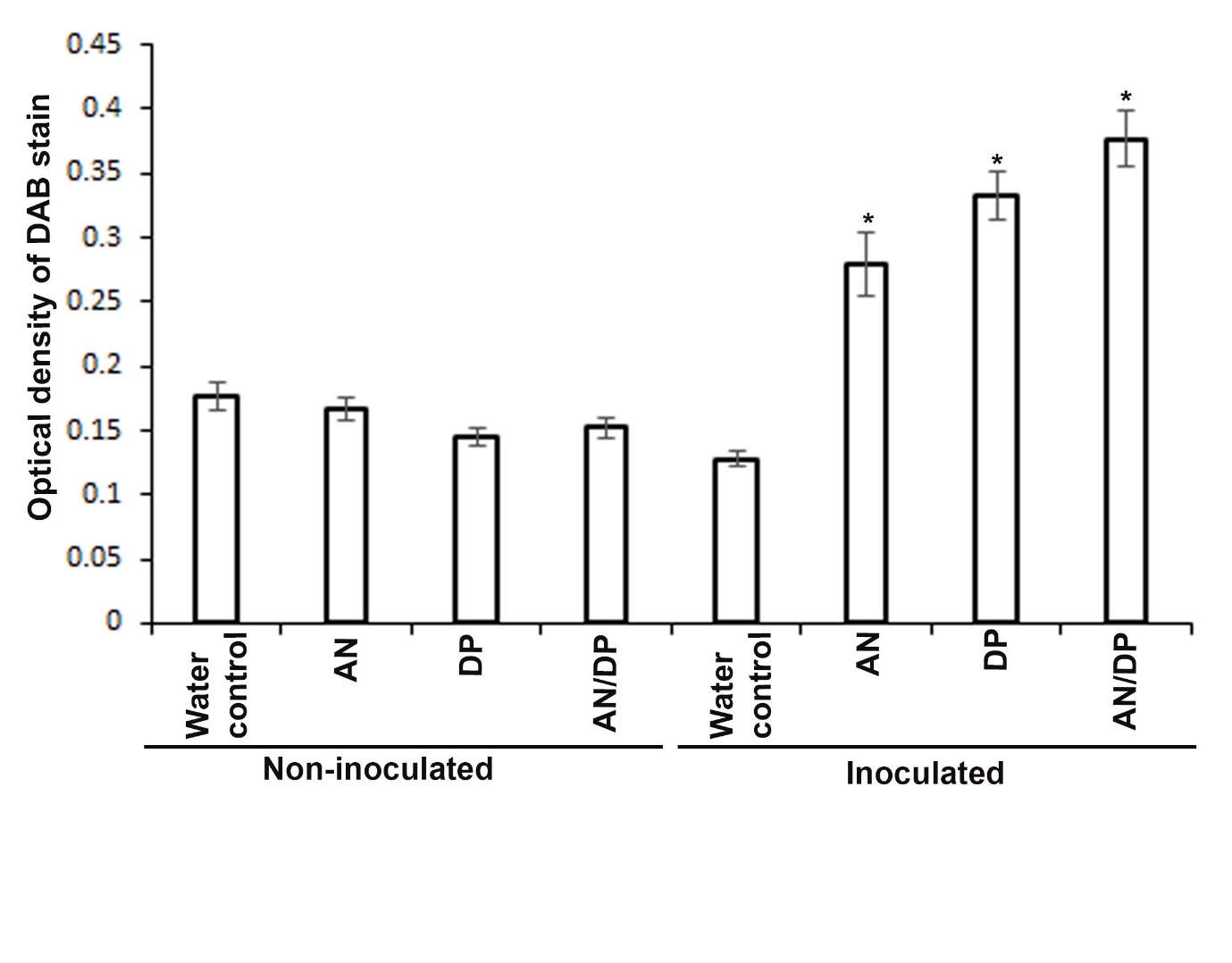
**Supplementary Figure 15.** Quantitation of ROS production at 12 hpi in roots of *A. thaliana* treated with seaweed extracts (AN, DP and AN/DP) and then inoculated with *P. cinnamomi*. DAB stain density in roots grown with indicated seaweed extracts and water as a control. * denotes significant difference compared to non-inoculated control of the respective treatment as well as the water treated control at *P*=0.05 according to Duncan’s multiple range test.
